# Design of strictly orthogonal biosensors for maximizing renewable biofuel overproduction

**DOI:** 10.1016/j.jare.2025.09.015

**Published:** 2025-09-10

**Authors:** Tong Wu, Dongli Yan, Sheng Lin, Ran Zhang, Yuhan Wang, Min Li, Shengzhu Yu, Xiaoyan Ma, Zhenya Chen, Yi-Xin Huo

**Affiliations:** a Key Laboratory of Molecular Medicine and Biotherapy, School of Life Science, Beijing Institute of Technology, No. 5 South Zhongguancun Street, Haidian District, Beijing 100081, China; b Tangshan Research Institute, BIT, No. 57, South Jianshe Road, Lubei District, Tangshan, Hebei 063000, China; c Beijing Jingtai Technology Co., Ltd., Beijing 100083, China

**Keywords:** Machine learning, Transcription factor, Strict orthogonality, Biofuel overproduction, Hydrogen bond

## Abstract

**Introduction:**

Transcription factors (TFs) activate transcriptional initiation by binding specific signal molecules (SMs), yet designing TFs to precisely target non-natural SMs remains challenging.

**Objectives:**

Using transcriptional activator BmoR as an example, a machine-learning based model named BT to predict three crucial residue regions (CRRs) was generated. This study aimed to achieve BmoR with strict SM orthogonality (SSO).

**Methods:**

Random Forest Algorithm was used to generate a model BT that pinpointed CRRs. The BmoR-SM complexes in the prediction dataset of Model BT were batch-simulated using a computational pipeline via Discovery Studio. Semi-rational engineering of the residues in the CRRs generated BmoR mutants with SSO, validated through MicroScale Thermophoresis (MST) affinity assays. The SSO-enabled BmoR-based biosensor was used to screen microbial overproducers for 3-L fed-batch fermentation.

**Results:**

The transcription activation effects of 245 TF-SM complexes were experimentally verified**,** providing the training and test dataset to generate a machine-learning based Model BT with 88.5 % accuracy. The binding between 5,700 BmoR mutants and four SMs was simulated by Discovery Studio, generating 22,800 complexes to output BmoR-SM hydrogen bond (BSH) counts. BSH counts combined with supplementary parameters to form a prediction dataset. The CRRs containing totally 36 residues were successfully predicted by Model BT. The CRRs were semi-rational modified to obtain BmoR mutants with SSO. The SSO-enabled BmoR-based biosensor effectively screened a strain yielding 12.6 g/L isopentanol.

**Conclusion:**

By demonstrating the dominant role of the BSH counts in TF-SM interactions and establishing a machine learning-guided framework for TF evolution, this work advances rational design principles for engineering TFs with precise molecular recognition, offering broad applications in synthetic biology and metabolic engineering.

## Highlights

• Machine-learning-based method was developed to engineer transcription factors (TFs).
• Generated BT model narrowed the TF mutagenesis region from 669 into 36 residues.
• TFs with strict signal molecule orthogonality were obtained.
• Biosensor screened out an isopentanol overproducer with recorded 12.6 g/L titer.

## Graphical abstract

**Figure:**
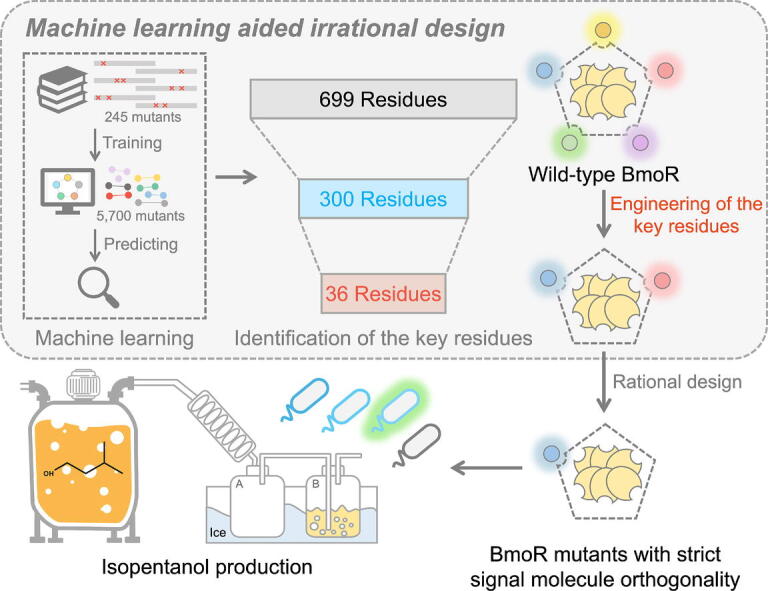

## Introduction

Transcription factors (TFs) regulate gene expression by sensing specific signal molecules (SMs) through binding energy [1,2], which governs their ability to form TF-SM complexes that interact with DNA regulatory regions to activate or repress transcription. Binding energy, influenced by molecular features of SMs and TF residues as well as their dynamic interactions, dictates transcriptional outcomes [3]. Lower binding energy promotes TF-SM complex formation under conditions where TF activators are activated or repressors are inhibited, facilitating RNA polymerase (RNAP) recruitment to upregulate transcription [4,5]. Conversely, reduced binding energy under repression of activators or activation of repressors favors the formation of TF-corepressor complexes that suppress transcription [5,6]. Base on this, an inverse relationship is established between binding energy and transcriptional activation or repression for a given RNAP-promoter system [6]. TF-SM binding relies on reversible non-covalent forces [7], including hydrogen bonds (HBs), hydrophobic interactions, electrostatic forces, van der Waals interactions and π-π stacking. HBs dominate binding stability, forming between SM hydroxyl/amino/carboxyl groups and complementary TF residues, requiring precise spatial alignment of donor–acceptor pairs [8]. Hydrophobic interactions stabilize TF active sites via nonpolar SM groups, while electrostatic forces depend on charge complementarity and environmental dielectric properties [9]. HB quantity directly governs binding strength and orthogonality, as complexes lacking HBs fail to persist despite other interactions [10]. Structural analogs of native SMs with HB-capable groups can mimic these bonds, triggering unintended transcriptional activation [11]. This highlights HB precision as a critical safeguard for maintaining regulatory accuracy and preventing off-target gene expression.

TF-based biosensors have gained significant traction in metabolic engineering due to their ability to convert SM signals into detectable outputs like fluorescence [12]. The biosensors enable high-throughput screening of enzyme libraries through fluorescence-activated cell sorting (FACS), support directed enzyme evolution via iterative mutagenesis and optimize metabolic pathways by dynamically coupling biosensor response with production networks [[13], [14], [15]]. Real-time monitoring of the metabolic flux further enhances their utility in balancing pathway efficiency [16,17]. However, limitations like low orthogonality, narrow detection range and poor sensitivity restrict the effectiveness, particularly in differentiating structurally similar compounds. Poor orthogonality may fail to distinguish structural analogs, generating false positives in enzyme or producer identification [18]. Despite ongoing efforts to engineer TF orthogonality, progress remains slow due to the labor-intensive nature of traditional methods. Binding energy between TFs and SMs governs transcriptional activation intensity, but designing ideal TF mutants remains challenging due to the multifactorial nature of binding energy and unclear parameter weighting, complicating targeted modifications for improved performance.

The TF BmoR from *Pseudomonas butanovora* activates transcription upon binding C2-C5 linear or branched-chain alcohols [19]. Its N-terminal, central, and C-terminal domains mediate alcohol sensing, σ^54^-RNAP interaction, and DNA binding respectively [20]. Engineered BmoR-based biosensors integrated with alcohol biosynthetic pathways enable fluorescence-driven screening of microbial overproducers [21]. Random mutagenesis of BmoR expanded detection capabilities, with mutants such as BmoR^T12N^ achieving broad detection range (0–200 mM), and BmoR^M94V/F272L^ or BmoR^S240P^ gaining orthogonality for isobutanol while retaining cross-reactivity with pentanols [22]. Pentanol isomers, valued as gasoline alternatives and flavor enhancers in fermented beverages, require precise monitoring due to their dual role, improving wine mouthfeel at optimal levels but causing adverse effects when overproduced [23,24]. The inherent SM promiscuity of BmoR complicates alcohol-specific detection, particularly in ethanol-dominated systems like wine fermentation where coexisting pentanols or butanols generate signal interference. This limitation underscores the need for refined biosensors capable of distinguishing structurally similar alcohols to ensure accurate metabolic monitoring and product quality control.

Current TF engineering relies on random mutagenesis coupled with high-throughput screening, while mutagenesis of the broad region often disrupts native TF functions while burying functional mutations among non-functional mutants during strain transformation, diminishing screening efficiency. Focusing mutagenesis on compact functional regions governing SM orthogonality, sensitivity, and activation range enhances the probability of obtaining ideal mutants. Identifying these pivotal regions remains vital for efficiently engineering TFs with high-performance [25].

Artificial intelligence (AI) drives advancements in synthetic biology and metabolic engineering, particularly the applications of machine learning in protein and enzyme engineering. AlphaFold of DeepMind exemplifies this progress by predicting protein structures with unprecedented accuracy [26,27]. Machine learning models excel in predicting protein–protein interactions using graph neural networks and accelerate enzyme engineering by integrating with directed evolution, enabling efficient screening of mutants with enhanced activity or stability [28]. These models also enable rational enzyme design by identifying structural or sequence features linked to improved performance, leveraging existing datasets to propose optimized mutants [29,30]. Coupling machine learning with high-throughput screening further streamlines mutant evaluation, minimizing experimental costs and accelerating the functional optimization [31,32].

**Fig. 1:**
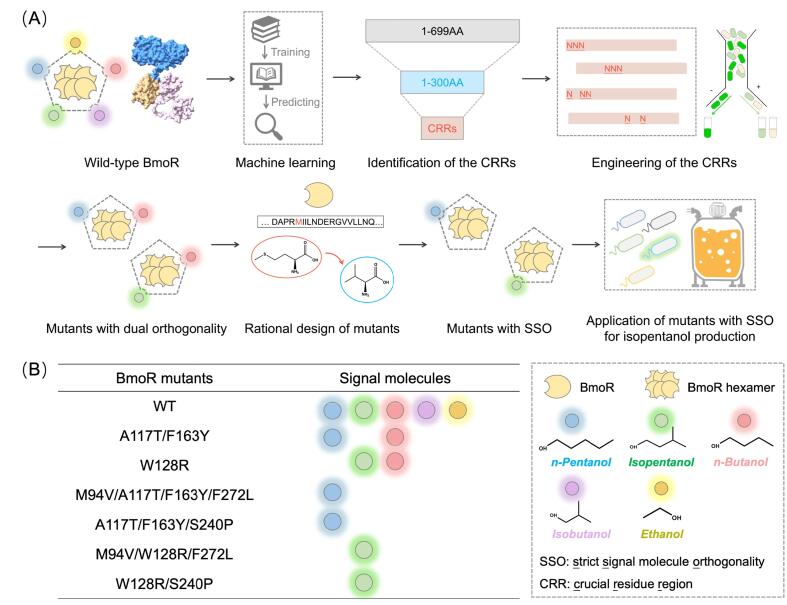
Machine-learning aided semi-rational design of BmoR with strict orthogonality. The crucial residues regions (CRRs) governing SM orthogonality of BmoR were identified via machine learning. BmoR achieved strict signal molecule orthogonality (SSO) via semi-rational design of the CRRs. (A) Overview of the workflow integrating machine learning and semi-rational design of BmoR with SSO and its application in isopentanol production. A machine learning-driven approach was established for engineering TF BmoR to achieve SSO. The approach pinpointed the CRRs, enabling semi-rational engineering of BmoR mutants with SSO. The BmoR mutants with SSO could apply to facilitate screening of isopentanol overproducers. (B) Representative BmoR mutants obtained in this study with remarkable SM selectivity. The circles with different colours represented different signal molecules (SMs). The number of the circles represented the response ability of BmoR mutants to SMs.

This study established a machine learning-driven approach for engineering TF BmoR to achieve strict SM orthogonality (SSO). Using BmoR as an example, a method to quantify the influence of transcriptional activation factors was developed, guiding the design of mutants with precise SM recognition. Random Forest Algorithm trained on full-length BmoR mutagenesis data (Supporting data1) to generate a model BT with 88.5 % prediction accuracy, revealing BmoR-SM hydrogen bond (BSH) counts were the primary determinant of activation. Discovery Studio simulations of 5,700 N-terminal mutants against four alcohol SMs generated 22,800 binding models, producing BSH counts combined with supplementary parameters to form a prediction dataset (Supporting data2). Model BT analysis of the prediction dataset pinpointed crucial residue regions (CRRs) governing SM orthogonality, enabling semi-rational engineering of BmoR mutants with SSO (Fig. 1A and B, Table 1). Affinity assays validated the SSO of mutants, while hexamer structure simulations revealed critical residues for SM discrimination. Application of an isopentanol-orthogonal BmoR-based biosensor facilitated screening of pentanol overproducers, yielding a record 12.6 g/L isopentanol titer. The approach integrates machine learning with molecular simulations to optimize TF engineering, demonstrating precision in resolving SM promiscuity and advancing biosensor-enabled metabolic overproduction.

**Table 1:** The parameters of BmoR mutants obtained in this study.

Parameters	BmoR mutants	Signal molecules			
		*n*-Pentanol	Isopentanol	*n*-Butanol	Isobutanol
Apparent *K*_m_ (mM)	WT	5.09	3.99	7.90	8.75
	A117T/F163Y	59.5	/	7.83	22.3
	W128R	/	∼5.68 × 10^15^	∼4.54 × 10^16^	63.3
	M94V/A117T/F163Y/F272L	∼1.96 × 10^16^	/	/	/
	A117T/F163Y/S240P	∼1.56 × 10^16^	/	/	/
	M94V/W128R/F272L	/	∼6.04 × 10^15^	/	/
	W128R/S240P	/	∼1.89 × 10^16^	/	/
Binding sites	WT	Asp172 Pro173 Phe210 Met248	Met248 Phe210	Arg211 Gln212 Asn259Glu261	Arg211 Gln212 Asn259
	A117T/F163Y	Pro173 Met248	/	Asp172	/
	W128R	/	Asp172 Pro173 Met248 Asn249	Asp172 Pro173 Asn249	/
	M94V/A117T/F163Y/F272L	Pro173 Arg250 Leu260	/	/	/
	A117T/F163Y/S240P	Arg250 Leu260	/	/	/
	M94V/W128R/F272L	/	Asp172 Pro173 Leu260	/	/
	W128R/S240P	/	Asp172 Pro173 Met248	/	/

## Materials and methods

### Strains, mediums and materials

*E. coli* XL10-Gold was used for plasmid construction, screening and construction of BmoR mutagenesis library. *E. coli* MG1655 was used for assessment of the tolerance to pentanols. LB medium (10 g/L tryptone, 5 g/L yeast extract and 10 g/L NaCl) was used for strain incubation and library screening. M9Y medium (33.9 g/L Na_2_HPO_4_, 15 g/L KH_2_PO_4_, 5 g/L NH_4_Cl and 2.5 g/L NaCl, 1 mM MgSO_4_, 0.1 mM CaCl_2_, 10 mg/L VB_1_, 4 g/L yeast extract and 40 g/L glucose) was used for fermentation experiments. The details of strains used in this study were depicted in Table S1.

### Homology modeling and molecular simulation

AUTODOCK 1.5.6 and Chimera*X* 1.4 were used for homology modeling and molecular docking of the wild type and BmoR mutants with SMs (*n*-pentanol, isopentanol, *n-*butanol or isobutanol). The tertiary structure of wild-type BmoR which was modelled by AlphaFold was used as the template to simulate the structures of BmoR mutants. The hexamer structure of wild-type BmoR was predicted by AlphaFold 2.0 [26] and AlphaFold 3.0 [33], respectively. All BmoR mutant models were evaluated by PROCHECK and all models had satisfactory quality with over 85 % of the residues in the most favored region of calculated z-scores. A grid box (10 × 17 × 8) which encompassed the binding pocket of BmoR was set as search space to explore suitable substrate-binding regions. The interactions between BmoR mutants and SMs were analyzed and showed in the corresponding figures.

### Machine learning method for BmoR

Data collection. Two kinds of dataset were collected in the study, including learning dataset containing training dataset and testing dataset, and predication dataset.

Learning dataset. The learning dataset included 245 BmoR mutants via error-prone PCR across the full-length protein of BmoR. The five parameters including BSH counts, mutation site-containing domain, mutation site-embedded secondary domain, mutation site-to-SBD distance, and residue characteristics pre-/post-mutation of the BmoR-SM complexes were integrated as inputs. The response values (0” or “1”) of BmoR mutants to four SMs were taken as outputs. In order to precisely locate the mutation site-containing domain, BmoR was partitioned into seven functionally distinct domains, including N-terminal SM non-binding domain (SNBD, residues 1–170), SM-binding domain (SBD, residues 171–260), N-terminal loop domain (LD, residues 261–325), hexamer domain in N-terminal (HND, residues 326–335), hexamer domain in AAA^+^ domain (residues HAD, 336–499), hexamer domain in C-terminal domain (HCD, 500–585), and C-terminal regulatory domain (CD, residues 586–669). A stratified split allocated 208 mutants in the learning dataset for model training and 37 mutants in the learning dataset for accuracy validation.

Prediction dataset. The prediction dataset included 5,700 BmoR mutants via saturation mutagenesis across the N-terminal SM-recognition region (residues 1–300). Discovery Studio was used for batch processing to generate 22,800 complexes, including 5,700 saturation mutants, each of which was paired with four SMs respectively, outputting BSH counts. BSHs counts, integrated with mutation site-containing domain, mutation site-embedded secondary domain, mutation site-to-SBD distance, and residue characteristics pre-/post-mutation, were used as input parameters for prediction. The details of the code program for batch processing were showed in Supporting information.

The above data was cleaned and selected to construct a data dictionary. Building a prediction dataset based on the data dictionary of the training dataset. Reading the training dataset, the prediction dataset, and assembling the tables. To transform the string variables into types suitable for machine learning (integer, float), using label encoding to map and transform the original string-type variables, including primary amino acids, mutated amino acids, structural domains, and secondary structures into integer types. x_train, y_train, x_pdt was obtained by assembling the tables.

The model variable named model 1 was defined as a decision tree, with a maximum depth of 5 and a random seed of 9. The fit and predict methods of model 1 was called for fitting and prediction, and the importance of the features was computed. Then, a canvas was set up and the decision tree was drawn to obtain y_pdt 1. The model variable model 1 was defined as a decision tree, with 500 estimators and a random seed of 9. The fit and predict methods of model 2 were called for fitting and prediction, and the importance of the features was computed. The decision tree was drawn to obtain y_pdt 2. The model variable model 1 was defined as a decision tree, with 500 estimators and a random seed of 9. The fit and predict methods of model 2 were called with the prediction result parameters defined as the orthogonality recognition of one of the four alcohols in the fit method. The PartialDependenceDisplay method from estimator was called to draw the PDP plot, thereby displaying the influence of the distribution of each independent variable on the prediction results.

### Establishment of the precise mutagenesis library

Three pairs of primers including three degenerate primers (named F-1, F-2 and F-3) and three normal primers (named F-4, F-5 and F-6) were designed and used to synthesize a fragment targeting the residues within the CRRs (Fig. S1). A pair of normal primers (named F-7, F-8) was designed and used to amplify a backbone using the plasmid pYH1 as the template. The above two PCR products were confirmed and purified via agarose gel electrophoresis. The purified products were digested by DpnI and then were assembled. The ligation product was transferred into 50 μL *E. coli* XL10-Gold competent cell, and a precise mutagenesis library was obtained. The details of plasmids and primers used in this study were described in Table S1 and S2, respectively. The construction process of plasmids used in this study was displayed in Supporting Information.

### High-throughput screening of the precise mutagenesis library

The mutagenesis library was pre-inoculated into 5 mL LB medium with 50 μg/mL carbenicillin and then cultured at 37 °C overnight. To screen BmoR mutants with orthogonal response towards *n*-pentanol, 50 μL of the seed culture was transferred into 5 mL LB medium which was supplemented with appropriate antibiotics and 10 mM *n*-pentanol, and the culture was then left at 30 °C for 8–10 h. Besides, another 50 μL of the seed culture was transferred into 5 mL LB medium which was supplemented with appropriate antibiotics and 10 mM isopentanol, and the culture was then left at 30 °C for 8–10 h to screen the mutants with orthogonal response towards isopentanol.

After obtaining the samples of the induced mutagenesis library (approximately 20,000 cells), two consecutive rounds of screening were conducted using FACS. Firstly, the sample containing wild-type BmoR-based biosensor was analyzed to determine the location of its fluorescence peak (P1) and the appropriate sorting voltage. Base on this, the samples of the mutagenesis library were sorted. In the first round of FACS, the bacteria from the top 1 % of the P3 region (approximately 1,000 cells) was collected and placed in 1 mL of antibiotic-free LB medium. After collection, 1 mL of the collected medium was added into 4 mL antibiotic-free LB medium, followed by incubation at 37 °C for 1 h. After that, 5 μL of 50 mg/mL carbenicillin was added, and the culture was incubated at 37 °C overnight.

Obtaining the library enriched in the first round of sorting, the same induction method was used for the second round of induction and FACS sorting. The second round of sorting used the fluorescence peak shift observed in the first round as a reference, and the top 1 % of the bacteria from the P3 region (approximately 1,000 cells) was collected. After collection, 4 mL of antibiotic-free LB medium was added to the collected bacterial culture, followed by incubation at 37 °C for 1 h. After incubation, 200 μL of the culture was spread on agar plates containing carbenicillin and incubated overnight at 37 °C. Approximately 1,000 single colonies were obtained, which had distinguishing GFP values when compared with the colonies containing wild-type BmoR-based biosensor.

During the sorting process, the fluorescence distribution was monitored using a BD FACS AriaII flow cytometer (BD Biosciences) with a 488 nm excitation laser and a 530/30 nm bandpass emission filter. A nozzle diameter of 70 μm was selected. The fluorescence and scatter signals were acquired on the following channels: Threshold (voltage 500 V), FITC (voltage 550 V), FSC (voltage 600 V), and SSC (voltage 400 V). The number of droplets and the sorting range were determined based on the population distribution and the scale of the mutation library. To mitigate the device errors and ensure the reliability of positive results, each library was sorted in three parallel groups, and the sorted cells were pooled for subsequent analysis.

The response values of the above colonies containing various BmoR mutants to *n*-pentanol, isopentanol, *n-*butanol or isobutanol were tested. The single colonies were pre-inoculated into 5 mL LB medium with 50 μg/mL carbenicillin and then cultured at 37 °C overnight. After that, 50 μL of the seed culture was transferred into 950 μL LB medium which was supplemented with appropriate antibiotics and 10 mM *n*-pentanol, isopentanol, *n-*butanol or isobutanol in 96-deep-well plates and the cultures were then left at 30 °C for 16 h. A microplate reader (BioTek Cytation 3) was used to detect OD_600_ and GFP values. The excitation and emission wavelengths were set as 470 and 510 nm, respectively. The fluorescence values were normalized as the GFP/OD_600_ values. Besides, 1 mL of the culture was centrifuged at 5,000 rpm for 5 min. The supernatant was discarded, and the sediment was then resuspended in 2 mL of the physiological saline. 5 μL of the culture was spotted onto glass slides for fluorescence microscopy (UOP 2100). Fifteen colonies which only responded to 10 mM *n*-pentanol or isopentanol were screened out. The *bmoRs* in the 15 colonies were amplified and sequenced to identify the mutation sites. Finally, the *bmoRs* in the five colonies were mutated.

### Gradient concentration assays

To obtain the response curves and measure the apparent *K*_m_ values of BmoR mutants towards SMs, the pentanol concentrations in 96-deep-well plates ranged between 0 and 60 mM, and butanols concentrations ranged between 0 and 100 mM. A microplate reader (BioTek Cytation 3) was used to detect OD_600_ and GFP values. The OD_600_ values needed to be subtracted from the OD_600_ value of the blank LB culture. The excitation and emission wavelengths were set as 470 and 510 nm, respectively. The fluorescence values were normalized as the GFP/OD_600_ values. Besides, the GFP/OD_600_ values of the strain containing the plasmid pYH7 was regarded as the background fluorescence to be subtracted. Apparent *K*_m_ values were estimated with Prism 9.5.0 through non-linear regression of the Michaelis-Menten equation. Finally, one or two effective mutants with orthogonal response towards *n*-pentanol or isopentanol were obtained.

### Competitive assays under mixed SMs

To verify the response of BmoR mutants with SM orthogonality towards SM mixtures, the ratio of *n*-pentanol, isopentanol, *n-*butanol or isobutanol in the culture was set as 1: 10 (10 mM: 100 mM),1: 8 (10 mM: 80 mM) 1: 6 (10 mM: 60 mM), 1: 4 (10 mM: 40 mM), 1: 2 (10 mM: 20 mM), 1: 1 (10 mM: 10 mM), 1: 0 (10 mM: 0 mM), 0: 1 (0 mM: 10 mM), 1: 1 (10 mM: 10 mM), 2: 1 (20 mM: 10 mM), 4: 1 (40 mM: 10 mM), 6: 1 (60 mM: 10 mM), 8: 1 (80 mM: 10 mM) or 10: 1 (100 mM: 10 mM). To confirm the insensitivity and orthogonality of BmoR mutants towards SM mixtures, the ratio of *n*-pentanol to isopentanol was set as 1: 6 (10 mM: 60 mM), 1: 4 (10 mM: 40 mM), 1: 2 (10 mM: 20 mM), 1: 1 (10 mM: 10 mM), 1: 0 (10 mM: 0 mM), 0: 1 (0 mM: 10 mM), 1: 1 (10 mM: 10 mM), 2: 1 (20 mM: 10 mM), 4: 1 (40 mM: 10 mM) or 6: 1 (60 mM: 10 mM) under the noise of 300 mM ethanol in the culture.

### The affinity determination by MST

Plasmids pWT-SUMO-BmoR-TEV-sfGFP-His, pWT-SUMO-BmoR^M94V/A117T/F163Y/F272L^-TEV-sfGFP-His, and pWT-SUMO-BmoR^M94V/W128R/F272L^-TEV-sfGFP-His were individually transformed into *E. coli* BL21(DE3). Single colonies were inoculated into 3 mL LB medium and cultured at 37 °C for 8–10 h to obtain seed cultures. Subsequently, 100 µL of the seed culture was transferred to 100 mL LB medium and grown at 37 °C until the OD_600_ value reached 0.8. 0.1 mM IPTG was added into the culture, and the culture was incubated at 16 °C, 170 rpm for 12–18 h. 50 mL centrifuge tubes were used to centrifuge the culture at 5,000 rpm for 15 min to collect the cell pellets. The pellets were then resuspended in 15 mL of the binding buffer, and the cells were disrupted using the ultrasonic cell crusher. The mixture was then centrifuged at 10,000 rpm for 30 min to collect the supernatant. The *K*_d_ values of the wild-type BmoR and the mutants towards four SMs were measured by the MicroScale Thermophoresis (MST, Nanotemper) from Wuhan Bio-lab Biotechnology Co., Ltd.

A 10 µL aliquot of the supernatant was diluted three folds with the assay buffer. The MST sample chamber was opened, and the tray was removed. The diluted protein sample was aspirated using a capillary and placed in the corresponding slot of the tray. The information of the protein samples and assay bugger was input, and the type of capillary was selected. The excitation power was set to “Auto”, and MST power was set to “High”. The concentration of the samples was adjusted using the assay buffer to 1/3 for BmoR^WT^, BmoR^M94V/A117T/F163Y/F272L^ and BmoR^M94V/W128R/F272L^. The samples were diluted to 100 µL/2000 µM using the assay buffer. Two eight-well strips were prepared and labeled from 1st to 16th. 10 µL of the assay buffer (1 % DMSO) was added into the 2nd-16th tubes, while 20 µL of the prepared sample was added to the 1st tube. 10 µL from the 1st tube was sequentially diluted into the 2nd-16th tubes. Subsequently, 10 µL of the lysate was added to each tube and mixed thoroughly. The mixed samples were incubated at 37 °C for 15 min before aspirating with a capillary to load into the sample tray. The software was started to begin the experiment.

### Screening of pentanol-tolerant strains and overproducers

To assess the tolerance of wild-type MG1655 to *n*-pentanol or isopentanol, the single colony of wild-type MG1655 was inoculated into 3 mL LB medium and cultured at 37 °C for 8–10 h to obtain the seed culture. 50 µL of the seed culture was transferred to 5 mL LB medium adding 0–100 mM *n*-pentanol or isopentanol and grown at 37 °C with 220 rpm for 12 h. A microplate reader (BioTek Cytation 3) was used to detect the OD_600_ values.

A library of *E. coli* MG1655-derived strains with different genomic large-fragment deletions ranging from 14 to 143 kb was constructed and stored [34]. To screen pentanol-tolerant strains, the strains from the library were inoculated into 3 mL LB medium and cultured at 37 °C for 8–10 h to obtain seed cultures. Subsequently, 50 µL of the seed culture was transferred to 5 mL LB medium in the presence of 20 mM *n*-pentanol or isopentanol and grown at 37 °C with 220 rpm for 12 h. A microplate reader (BioTek Cytation 3) was used to detect the OD_600_ values. The pentanol-tolerant strains were obtained with the higher OD_600_ values.

To screen an isopentanol overproducer from the pentanol-tolerant strains, plasmids pWT-SCDA*BCD containing genes *alsS*, *ilvC*, *ilvD* and *leuA^G462D^BCD*, and pWT-B*-LBD*D containing genes *tdcB*, *kivD^V461A/M538A^*, *yqhD* and the BmoR^M94V/W128R/F272L^-based biosensor were introduced into wild-type MG1655 or the pentanol-tolerant strains. The single colonies were pre-inoculated into 3 mL LB medium with associated antibiotics at 37 °C, 220 rpm for 8–10 h. Then, 50 μL of the culture was inoculated into 950 μL M9Y medium (2 g/L yeast extract, 10 % glucose) with 1 mM IPTG at 30 °C with 220 rpm for 24 h. Microplate reader (BioTek Cytation 3) was used to detect the OD_600_ and GFP values. The excitation and emission wavelengths were set as 470 and 510 nm, respectively. The isopentanol overproducer was obtained with the highest GFP/ OD_600_ values. The whole screening process was illustrated in Fig. S2.

### Shake-flask fermentation of the isopentanol overproducer

Plasmids pWT-SCDA*BCD containing genes *alsS*, *ilvC*, *ilvD* and *leuA^G462D^BCD*, and pWT-LBD*D containing genes *tdcB*, *kivD^V461A/M538A^* and *yqhD* were introduced into wild-type MG1655 or Δ33. The single colonies were pre-inoculated into 3 mL LB medium with associated antibiotics at 37 °C for 8–10 h. Then, 200 μL of the culture was inoculated into 20 mL M9Y (4 g/L yeast extract, 40 % glucose) with 1 mM IPTG in 250 mL screw cap conical flask and left at 30 °C with 220 rpm. Samples were taken every 12 h for OD_600_ measurement and isopentanol detection.

Agilent 6890 GC chromatograph which was equipped with flame ionization detector (Agilent Technologies, CA, USA) was used for detecting fermentation samples. The alcohols were separated by a DB-FFAP capillary column (30 m × 0.32 mm × 0.25 μm, Agilent Technologies). For analysis of samples, the GC oven temperature was initially held at 80 °C for 3 min, increased with a gradient of 115 °C/min until 230 °C, and kept at 230 °C for an additional 1 min. Nitrogen was used as the carrier gas. The injector and detector were maintained at 250 and 280 °C, respectively. Supernatant (0.2 μL) was sampled and injected at a split ratio of 1: 30 and isopropanol was used as internal standard.

### Fed-batch fermentation of the isopentanol overproducer

To evaluate the isopentanol-producing potential of the isopentanol overproducer, the fermentation was performed in a 3-L bioreactor with 1 L working volume. The culture medium containing 20 g/L glucose, 3 g/L (NH_4_)_2_SO_4_, 14.6 g/L K_2_HPO_4_, 4 g/L KH_2_PO_4_, 2.2 g/L sodium citrate, 8 g/L yeast extract, 1.25 g/L MgSO_4_·7H_2_O, 0.05 g/L carbenicillin, 0.05 g/L kanamycin, 1 mL/L trace metal solution and 0.5 mM IPTG was used for fed-batch fermentation. Trace metal solution contained 14.1 g EDTA, 2.5 g CoCl_2_·6H_2_O, 15 g MnCl_2_·4H_2_O, 1.5 g CuCl_2_·2H_2_O, 3 g H_3_BO_3_, 2.1 g Na_2_MoO_4_·2H_2_O, 33.8 g Zn (CH_3_COO)_2_·2H_2_O and 80 g FeCl_3_·6H_2_O per liter. During the cultivation period, 1 L stock solution containing 500 g/L glucose, 1.25 g/L MgSO_4_·7H_2_O, 0.05 g/L carbenicillin, 0.05 g/L kanamycin and 0.5 mM IPTG was fed to the batch culture.

The bioreactor was inoculated with 5 % of overnight preculture and the cells grown at 37 °C with 1 vvm of air flow rate and 600 rpm of stirrer speed for 3.5 h. Then, the temperature was changed to 30 °C to induce the expression of the enzymes in isopentanol production pathway. The pH was controlled at 6.8 by automatic addition of ammonia solution (25 %). The evaporated isopentanol was condensed by a condenser and subsequently the generated liquid isopentanol flowed into collection bottle A which was cooled with ice. The residual uncondensed isopentanol was collected into bottle B which containing 800 mL water and was also cooled with ice. The samples were taken to determinate the biomass, glucose concentration and isopentanol titer.

## Results

### Machine learning-driven identification of the CRRs for SM orthogonality

**Fig. 2:**
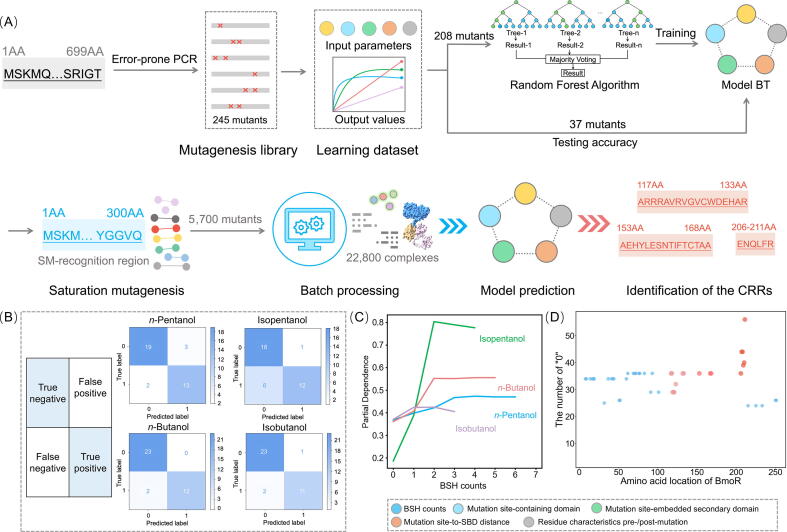
Machine learning-driven identification of the CRRs for SM orthogonality. (A) The whole process of machine learning-driven identification of the CRRs for SM orthogonality. Dataset construction. A training dataset of 245 error-prone PCR mutants was generated, and the response values to four SMs were taken as outputs. Molecular simulations quantified five structural parameters (BSH counts, mutation site-containing domain, mutation site-embedded secondary domain, mutation site-to-SBD distance, residue characteristics pre-/post-mutation) paired with outputs. Random Forest Algorithm trained on 208 mutants to generate Model BT. 37 mutants were used to test the accuracy of Model BT. Model BT predicted the outputs of 5,700 saturation mutants across the N-terminal SM-recognition region (residues 1–300) to identify the CRRs. Discovery Studio was used for batch processing to generate 22,800 complexes, including the 5,700 saturation mutants, each of which was paired with four SMs respectively. (B) Testing the accuracy of Model BT. The confusion matrix was used to evaluate the errors of machine-learning-predicted classifications. The real experimental response values to four SMs of 37 mutants were compared with the outputs predicted by Model BT, and the comparison results displayed in the confusion matrix. The main-diagonal elements (True positive and True negative) represented the results of the predicted outputs being consistent with the real response value, while the back-diagonal elements (False positive and False negative) represented the results of the predicted outputs being opposite to the real response value. (C) The corresponding partial dependence plots of BSH counts in BmoR-SM complexes. These plots quantified the marginal effect of BSH counts on the response of BmoR towards four SMs. Curves revealed BSH counts significantly influenced the response of BmoR towards four SMs. (D) CRRs identification. Model BT predicted the response values of 5,700 saturation mutants to four SMs. A binary output of “0” indicated residues whose mutagenesis substantially altered BmoR-SM binding, with higher “0” frequency correlating with greater functional impact. Residues exhibiting significant binding influence and their neighbours were classified in the CRRs. Red circles highlighted CRR-localized residues, while blue circles denoted residues in non-critical regions. (For interpretation of the references to colour in this figure legend, the reader is referred to the web version of this article.)

First, a learning dataset was established for the construction of Model BT. The wild-type BmoR-based biosensor exhibited promiscuous recognition of four SMs (*n*-pentanol, isopentanol, *n*-butanol, and isobutanol, Fig. S3 and S4, Table 1). The *K*_d_ values of wild-type BmoR towards these four SMs were 0.106 μM, 0.0938 μM, 1.25 μM and 11.8 μM, respectively (Fig. S5, Table 2). To dissect the determinants of the SM orthogonality of BmoR, a learning dataset was constructed by generating 245 BmoR mutants via error-prone PCR across the full-length protein. The response of each mutant to the four SMs was quantified as GFP/OD_600_, with a ≥ 25 % increase over background defined as a positive output “1”, and below this threshold as negative “0”. Molecular simulations of mutated BmoR-SM complexes were performed to analyze BSH counts, mutation site-containing domain, mutation site-embedded secondary domain, mutation site-to-SBD distance, and residue characteristics pre-/post-mutation. These parameters were integrated as inputs, paired with binary outputs, forming a learning dataset of 245 mutants. A stratified split allocated 208 mutants for model training and 37 mutants for accuracy validation (Fig. 2A).

**Table 2:** The affinity of BmoR mutants towards SMs.

Fusion of BmoR with sfGFP	SMs	*K*_d_ (μM)	*K*_d_ Confidence (μM)	Signal/Noise
WT	*n*-Pentanol	0.106	±0.077	7.86
	Isopentanol	0.0938	±0.0339	15.9
	*n*-Butanol	1.25	±0.51	9.46
	Isobutanol	11.8	±5.8	6.83
M94V/A117T/F163Y/F272L	*n*-Pentanol	322	±177	11.5
	Isopentanol	/	/	/
	*n*-Butanol	/	/	/
	Isobutanol	/	/	/
M94V/W128R/F272L	*n*-Pentanol	/	/	/
	Isopentanol	0.0261	± 0.0202	14.0
	*n*-Butanol	/	/	/
	Isobutanol	/	/	/

Second, Model BT was developed and validated using the Random Forest Algorithm, which was trained on a dataset comprising 208 mutants (Fig. S6). Model BT demonstrated robust predictive performance across the testing dataset comprising 37 mutants. Specifically for the response values of the mutants towards *n*-pentanol, the model achieved 86.5 % accuracy (32/37 mutants), with the experimental response values of 13 true positives and 19 true negatives aligning precisely with predicted response values (Fig. 2B). Extended validation incorporating the response values of the mutants towards isopentanol, *n*-butanol, and isobutanol revealed enhanced model generalizability, culminating in an overall prediction accuracy of 88.5 % across all tested mutants. The Root Mean Squared Error of the model to test the response values of the mutants towards *n*-pentanol, isopentanol, *n*-butanol or isobutanol was 0.367, 0.435, 0.232 or 0.285, respectively. The partial dependence analysis of Model BT systematically quantified the parameter sensitivity landscape, revealing how input parameters (e.g., BSH count, mutation site-containing domain) nonlinearly modulated output values (Fig. 2C and S7). Strikingly, BSH counts exhibited statistically significantly positive coordination with output values. These highlighted BSH formation as a critical driver of biosensor orthogonality. To further probe this, the N-terminal SM-recognition region (residues 1–300) was focused, hypothesizing that mutagenesis in this region would modulate BSH formation and alter SM selectivity.

Third, three critical orthogonality-defining regions were identified. 5,700 saturation mutants were designed across the N-terminal SM-recognition region (residues 1–300) and implemented a computational pipeline to batch-simulate 22,800 BmoR-SM complexes via Discovery Studio, extracting BSH counts. The 22,800 complexes included the 5,700 saturation mutants, each of which was paired with four SMs respectively. Integrating BSH counts with mutation site-containing domain, mutation site-embedded secondary domain, mutation site-to-SBD distance, and residue characteristics pre-/post-mutation, Model BT predicted the response values of 5,700 saturation mutants to four SMs. A binary output of “0” indicated residues whose mutagenesis substantially altered BmoR-SM binding, with higher “0” frequency correlating with greater functional impact. Residues exhibiting significant binding influence and their neighbours were classified in the CRRs. Base on these, three CRRs were identified, including 117th-133rd AA, 153rd-168th AA and 206th-211th AA (Fig. 2D), whose saturation mutagenesis had significant effect on the output values compared to the saturation mutagenesis in other regions. In the binding sites of wild-type BmoR (including Asp172, Pro173, Phe210, Met248, Arg211, Gln212, Asn259, and Glu261) towards SMs (Fig. S3C and D, Table 1), although only Phe210 and Gln212 were situated within the CRRs, mutations occurring at non-binding sites within the CRRs could break the HB networks, thereby substantially changing the SM orthogonality. Therefore, engineering of the residues in the CRRs could enable the development of mutants with strict orthogonality for distinct SMs.

### Engineering of the CRRs for screening mutants with dual orthogonality

**Fig. 3:**
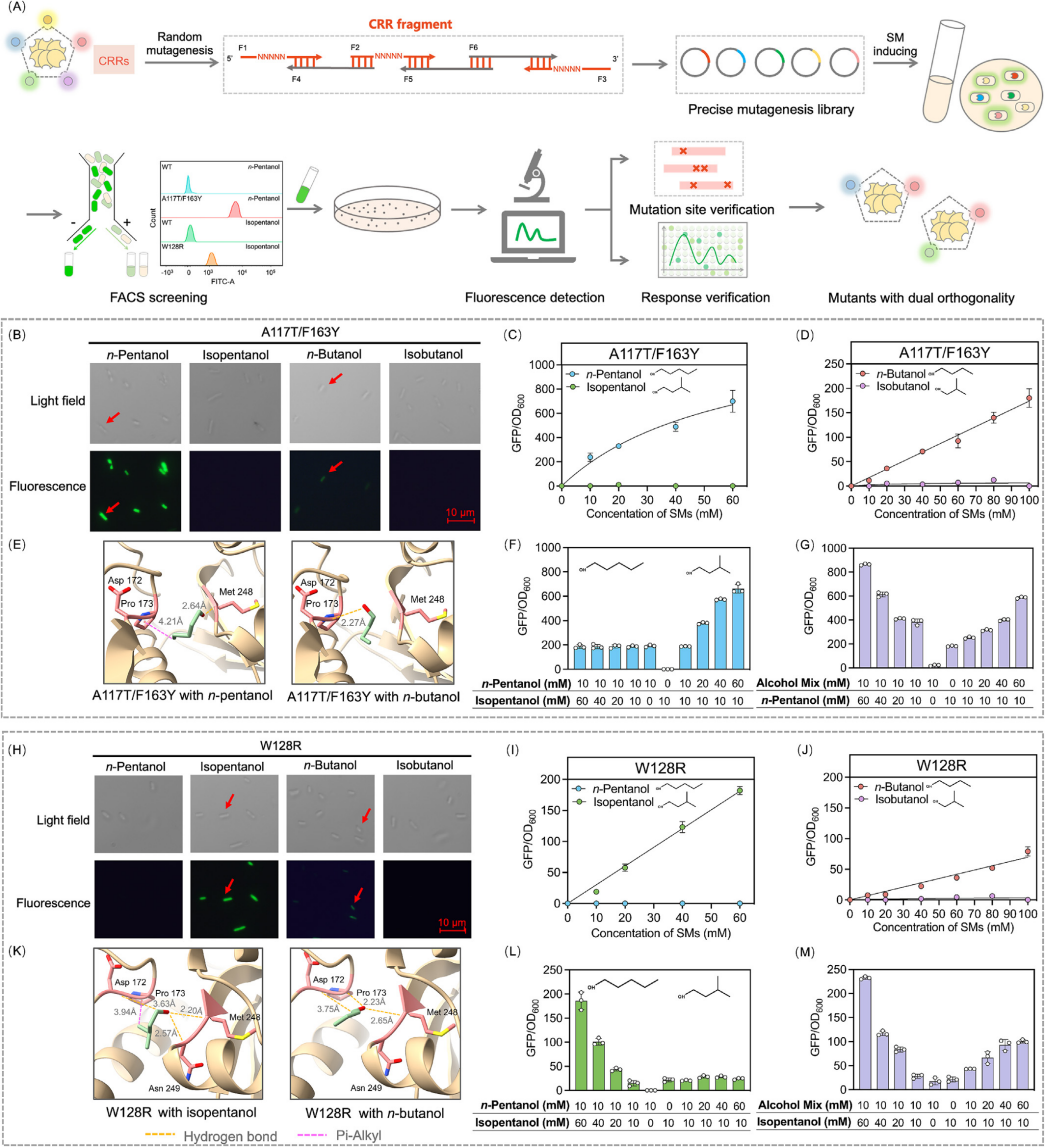
Engineering of the CRRs for screening mutants with dual orthogonality. (A) The whole engineering and screening process of the mutagenesis library of the CRRs. A precise mutagenesis library targeting the residues within the CRRs was constructed using degenerate primers. High-throughput screening of mutagenesis library was conducted through two rounds of FACS. The mutation sites and the response values to four SMs of the obtained mutants were verified. (B) Green fluorescence of the strain harboring BmoR^A117T/F163Y^ in presence of 10 mM SMs. (C) Response curves of BmoR^A117T/F163Y^ towards *n*-pentanol or isopentanol. (D) Response curves of BmoR^A117T/F163Y^ towards *n*-butanol or isobutanol. (E) Molecular simulations of BmoR^A117T/F163Y^ with *n*-pentanol or *n*-butanol. (F) Maintaining the concentration (10 mM) of *n*-pentanol or isopentanol, and increasing the concentration of *n*-pentanol or isopentanol with a gradient (0–60 mM) to confirm the orthogonality of BmoR^A117F/F163Y^ towards *n*-pentanol and *n*-butanol. (G) Maintaining the concentration (10 mM) of the SM mixture (isopentanol, *n*-butanol and isobutanol) or *n*-pentanol, and increasing the concentration of the SM mixture or *n*-pentanol with a gradient (0–60 mM) to confirm the orthogonality of BmoR^A117F/F163Y^ towards *n*-pentanol and *n*-butanol. (H) Green fluorescence of the strain harboring BmoR^W128R^ in presence of 10 mM SMs. (I) Response curves of BmoR^W128R^ towards *n*-pentanol or isopentanol. (J) Response curves of BmoR^W128R^ towards *n*-butanol or isobutanol. (K) Molecular simulations of BmoR^W128R^ with *n*-pentanol or isopentanol. (L) Maintaining the concentration (10 mM) of *n*-pentanol or isopentanol and increasing the concentration of *n*-pentanol or isopentanol with a gradient (0–60 mM) to confirm the orthogonality of BmoR^W128R^ towards isopentanol and *n*-butanol. (M) Maintaining the concentration (10 mM) of the SM mixture (*n*-pentanol, *n*-butanol and isobutanol) or isopentanol, and increasing the concentration of the SM mixture or isopentanol with a gradient (0–60 mM) to confirm the orthogonality of BmoR^W128R^ towards isopentanol and *n*-butanol. Values and error bars represent mean and s.d. (n = 3), respectively. (For interpretation of the references to colour in this figure legend, the reader is referred to the web version of this article.)

A precise mutagenesis library was constructed, systematically targeting the residues within the CRRs. The comprehensive design and screening workflow was detailed in Fig. 3A. Through two rounds of FACS, a strain harboring BmoR^A117T/F163Y^ was isolated, which exhibited robust green fluorescence in the presence of 10 mM *n*-pentanol but only a negligible response to 10 mM isopentanol (Fig. 3B). To characterize its SM orthogonality, gradient concentration assays with *n*-pentanol and isopentanol were performed to determine the apparent *K*_m_ values. As shown in Fig. 3C, the GFP/OD_600_ of BmoR^A117T/F163Y^ reached 699 ± 90 when supplemented with 60 mM *n*-pentanol, whereas no significant response was detected with isopentanol, confirming its exclusive sensitivity to *n*-pentanol concentration changes. Quantitative analysis revealed an apparent *K*_m_ value of 59.5 mM for *n*-pentanol (Table 1). Cross-reactivity testing against structural analogs *n*-butanol and isobutanol showed that BmoR^A117T/F163Y^ preferentially recognized *n*-butanol over isobutanol (Fig. 3D). Molecular simulations of the mutated BmoR-SM complexes, as shown in Fig. 3E, disclosed specific interactions. Specifically, *n*-pentanol established one HB between the hydrogen atom of its hydroxyl group and Met248 of the mutant. Meanwhile, a Pi-alkyl interaction occurred between the alkyl chain of *n*-pentanol and Pro173. For *n*-butanol, one HB was formed between its hydroxyl hydrogen and Asp172 of the mutant. In contrast, no stable interactions were formed in the mutant with isopentanol or isobutanol, explaining the discriminatory response mechanism of BmoR^A117T/F163Y^.

To validate the SM orthogonality of BmoR^A117T/F163Y^ towards *n*-pentanol and *n*-butanol, and assess potential competitive binding with structural analogs, the response to binary SM mixtures was systematically measured containing *n*-pentanol and its isomer isopentanol across varying molar ratios. The GFP/OD_600_ of BmoR^A117T/F163Y^ increased proportionally with the *n*-pentanol fraction in the mixtures, peaking at 664 ± 36 when the ratio reached 6: 1 (60 mM: 10 mM). In contrast, no significant change in fluorescence was observed as isopentanol concentration increased alone (Fig. 3F). These findings indicated that *n*-pentanol and isopentanol did not compete for binding to BmoR^A117T/F163Y^, and the mutant maintained exclusive orthogonality for *n*-pentanol even in the presence of its isomer. Parallel experiments with four additional SM mixtures revealed that GFP/OD_600_ increased exclusively with rising concentrations of *n*-pentanol or *n*-butanol across all tested combinations (Fig. 3G and S8). No activation was detected in response to isopentanol or isobutanol. These results demonstrated BmoR^A117T/F163Y^ as a dual-orthogonal mutant responsive to *n*-pentanol and *n*-butanol.

Subsequently, a mutant BmoR^W128R^ was isolated, which showed strong responsiveness by 10 mM isopentanol while remained unresponsive to 10 mM *n*-pentanol (Fig. 3H). To quantify its SM selectivity, gradient concentration assays revealed concentration-dependent activation by isopentanol with an apparent *K*_m_ of 58.2 mM, whereas *n*-pentanol elicited no detectable response (Fig. 3I). Cross-reactivity testing further demonstrated preferential recognition of BmoR^W128R^ towards *n*-butanol over its structural analog isobutanol (Fig. 3J). Molecular simulations uncovered distinct binding mechanisms governing SM selectivity, where isopentanol engaged Asp172, Met248 and Asn249 in BmoR^W128R^ via HBs and formed one Pi-alkyl interaction with Pro173. Besides, *n*-butanol interacted with Asp172, Pro173, and Asn249 in BmoR^W128R^ through HBs (Fig. 3K).

To validate the isopentanol and *n*-butanol orthogonality of BmoR^W128R^ and assess its binding competition with structurally SMs, response experiments were conducted by supplementing cultures with mixed SMs. The GFP/OD_600_ of BmoR^W128R^ exhibited dose-dependent activation correlating with isopentanol enrichment, peaking at 186 ± 18 when the ratio of *n*-pentanol and isopentanol reached 1: 6 (10 mM: 60 mM). Conversely, increasing *n*-pentanol proportions failed to elicit measurable responses (Fig. 3L), confirming the absence of competitive binding between these two SMs and the preserved isopentanol selectivity of BmoR^W128R^ under mixed SM conditions. Parallel experiments with four additional SM combinations revealed selective activation patterns, where GFP/OD_600_ elevations occurred only with isopentanol or *n*-butanol enrichment (Fig. 3M and S9), solidifying BmoR^W128R^ as a dual-orthogonal mutant responsive to isopentanol and *n*-butanol.

### Rational design of mutants with strict orthogonality

**Fig. 4:**
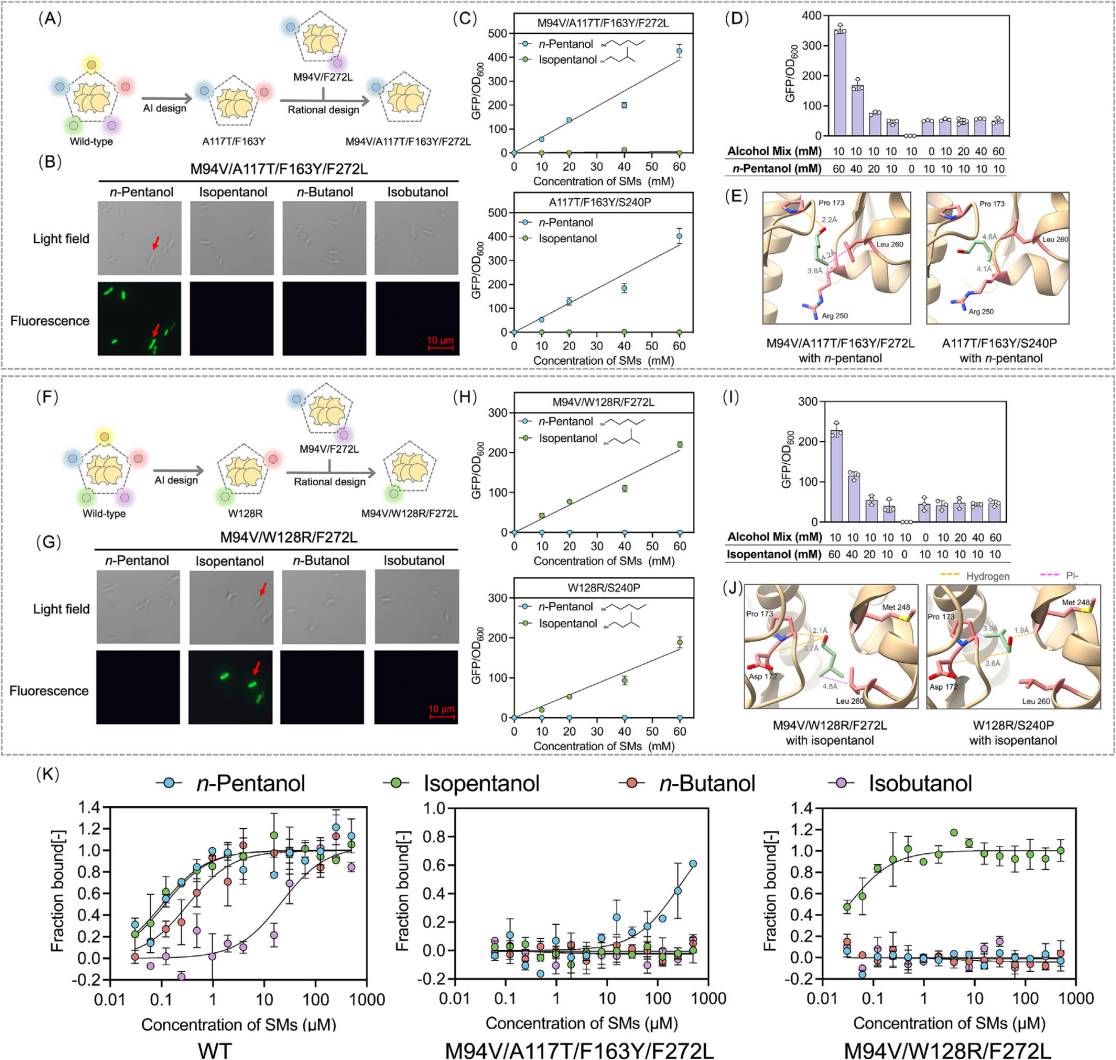
Rational design of mutants with strict orthogonality. (A) The rational design process of BmoR^A117T/F163Y^. The mutation sites M94V/F272L or S240P could enable BmoR to selectively sense isobutanol over *n*-butanol while retain pentanol responsiveness. These mutations were individually introduced into BmoR^A117T/F163Y^ to suppress its *n*-butanol sensitivity and achieve strict *n*-pentanol orthogonality. (B) Green fluorescence of the strain harboring BmoR^M94V/A117T/F163Y/F272L^ in presence of 10 mM SMs. (C) Response curves of BmoR^M94V/A117T/F163Y/F272L^ or BmoR^A117T/F163Y/S240P^ towards *n*-pentanol or isopentanol. (D) Maintaining the concentration (10 mM) of the SM mixture (isopentanol, *n*-butanol and isobutanol) or *n*-pentanol, and increasing the concentration of the SM mixture or *n*-pentanol with a gradient (0–60 mM) to confirm the orthogonality of BmoR^M94V/A117T/F163Y/F272L^ towards *n*-pentanol. (E) Molecular simulations of BmoR^M94V/A117T/F163Y/F272L^ or BmoR^A117T/F163Y/S240P^ with *n*-pentanol. (F) The rational design process of BmoR^W128R^. (G) Green fluorescence of the strain harboring BmoR^M94V/W128R/F272L^ in presence of 10 mM SMs. (H) Response curves of BmoR^M94V/W128R/F272L^ or BmoR^W128R/S240P^ towards *n*-pentanol or isopentanol. (I) Maintaining the concentration (10 mM) of the SM mixture (*n*-pentanol, *n*-butanol and isobutanol) or isopentanol, and increasing the concentration of the SM mixture or isopentanol with a gradient (0–60 mM) to confirm the orthogonality of BmoR^M94V/W128R/F272L^ towards isopentanol. (J) Molecular simulations of BmoR^M94V/W128R/F272L^ or BmoR^W128R/S240P^ with isopentanol. (K) The fraction bound fitting graphs of wild-type BmoR, BmoR^M94V/A117T/F163Y/F2727L^ and BmoR^M94V/W128R/F2727L^ towards four SMs. Values and error bars represent mean and s.d. (n = 3), respectively. (For interpretation of the references to colour in this figure legend, the reader is referred to the web version of this article.)

To create mutants with strict orthogonality towards either *n*-pentanol or isopentanol, BmoR^A117T/F163Y^ and BmoR^W128R^ were rationally modified to remove their responsiveness to *n*-butanol. Building on the previous study that BmoR^M94V/F272L^ and BmoR^S240P^ selectively sensed isobutanol over *n*-butanol while retaining pentanol responsiveness [22], it was hypothesized that combining these mutations would suppress *n*-butanol sensitivity in the dual-orthogonal mutants (Fig. 4A). The resulting mutants BmoR^M94V/A117T/F163Y/F272L^ and BmoR^A117T/F163Y/S240P^ exhibited exclusive *n*-pentanol responsiveness. Unlike their parental BmoR^A117T/F163Y^ (Fig. 3C and D), both mutants showed dose-dependent GFP/OD_600_ increased strictly correlating with *n*-pentanol concentrations (Fig. 4B and C), with no detectable responses to structural analogs. Competitive assays under mixed SM conditions further validated the unwavering *n*-pentanol orthogonality of BmoR^M94V/A117T/F163Y/F272L^ (Fig. 4D and S10). Molecular simulations revealed mechanistic underpinnings where *n*-pentanol formed one HB with Pro173 and two Pi-alkyl interactions with Arg250 and Leu260 in BmoR^M94V/A117T/F163Y/F272L^, while engaging two HBs in BmoR^A117T/F163Y/S240P^ (Fig. 4E). Notably, the complete absence of interactions with isopentanol, *n*-butanol or isobutanol in both mutants directly correlated these binding patterns to their stringent *n*-pentanol selectivity.

Next, the same mutation sites were introduced into BmoR^W128R^ and validated their functional impact (Fig. 4F). The engineered mutants BmoR^M94V/W128R/F272L^ and BmoR^W128R/S240P^ demonstrated exclusive isopentanol orthogonality, as evidenced by dose-dependent GFP/OD_600_ increased strictly proportional to isopentanol concentrations (Fig. 4G and H). Competitive assays under mixed SM conditions confirmed the unwavering isopentanol selectivity of BmoR^M94V/W128R/F272L^ even in the presence of structural analogs (Fig. 4I and S11). Molecular simulations revealed distinct interactions where isopentanol formed two HBs and one Pi-alkyl interaction with Asp172, Pro173 and Leu260 or Met248 in both mutants (Fig. 4J), contrasting with the complete absence of binding to *n*-butanol.

Moreover, the *K*_d_ values of the mutants with SSO were measured. The *K*_d_ value of BmoR^M94V/A117T/F163Y/F272L^ for *n*-pentanol was 322 μM (Fig. S12). The *K*_d_ value of BmoR^M94V/W128R/F272L^ for isopentanol was 0.0261 μM (Fig. S13, Table 2). Fnorm and fraction-bound analyses conclusively demonstrated exclusive binding of *n*-pentanol or isopentanol to their respective mutants without cross-reactivity to analogs (Fig. 4K). Collectively, the rationally designed mutants BmoR^M94V/A117T/F163Y/F272L^, BmoR^A117T/F163Y/S240P^, BmoR^M94V/W128R/F272L^, and BmoR^W128R/S240P^ achieved precise discrimination between pentanols and their analogs butanols through structure-guided molecular recognition.

### Robust orthogonality validation against ethanol interference

**Fig. 5:**
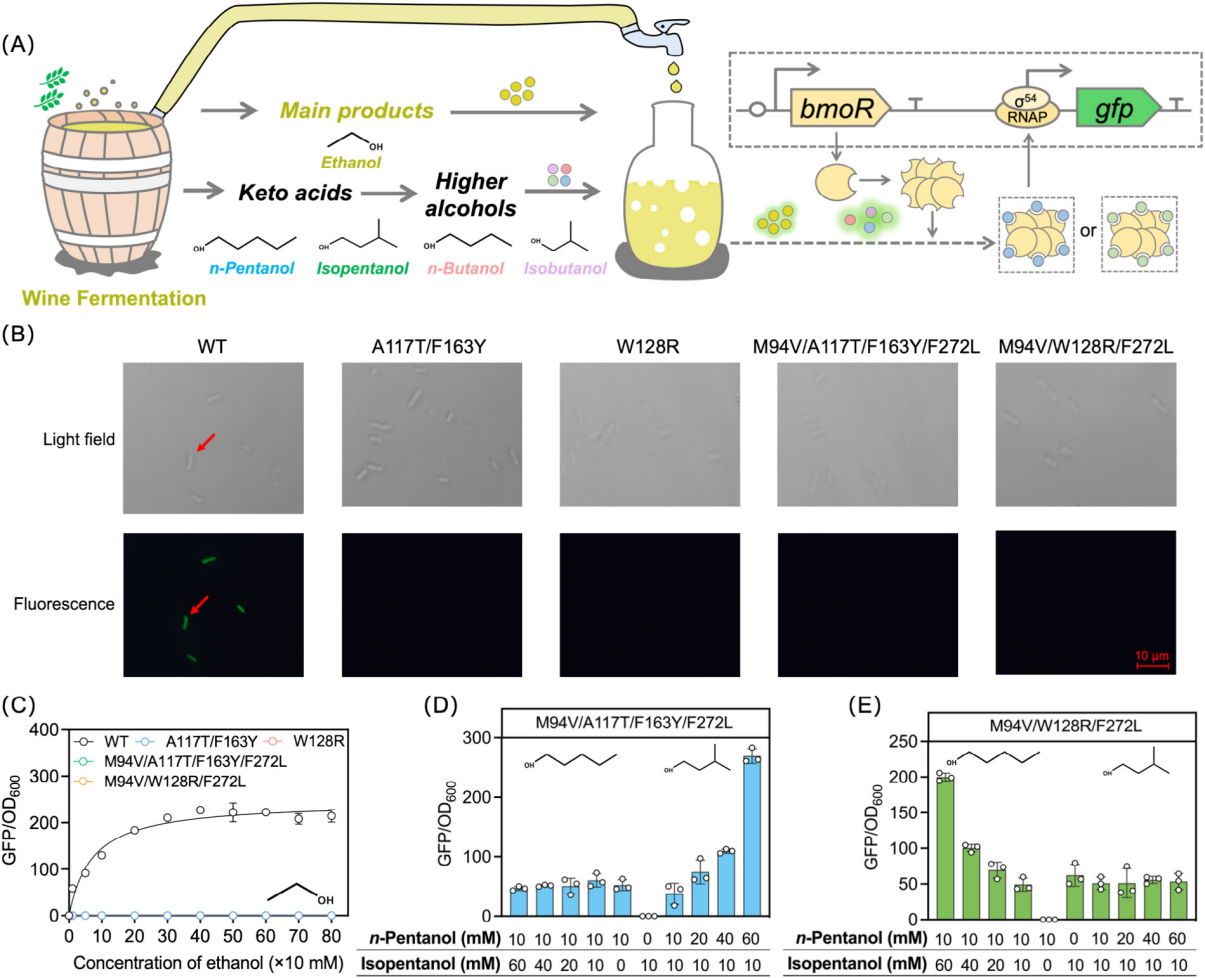
Robust orthogonality validation against ethanol interference. (A) The whole detective process of the flavor compounds in wine fermentation. The by-products including *n*-pentanol, isopentanol, *n*-butanol and isobutanol influenced the mouthfeel of the wine. SSO-enabled BmoR-based biosensor could monitor the *n*-pentanol or isopentanol in real time in wine fermentation. (B) Green fluorescence of the strains harboring different BmoR mutants in presence of 100 mM ethanol. (C) Response curves of BmoR mutants towards ethanol. (D) Maintaining the concentration (10 mM) of *n*-pentanol or isopentanol, and increasing the concentration of *n*-pentanol or isopentanol with a gradient (0–60 mM) under the noise of 300 mM ethanol to confirm the orthogonality of BmoR^M94V/A117T/F163Y/F272L^ towards *n*-pentanol. (E) Maintaining the concentration (10 mM) of *n*-pentanol or isopentanol, and increasing the concentration of *n*-pentanol or isopentanol with a gradient (0–60 mM) under the noise of 300 mM ethanol to confirm the orthogonality of BmoR^M94V/W128R/F272L^ towards isopentanol. Values and error bars represent mean and s.d. (n = 3), respectively. (For interpretation of the references to colour in this figure legend, the reader is referred to the web version of this article.)

Given that ethanol is a major product in wine fermentation, it was essential to validate the orthogonality of the newly generated mutants towards pentanols under ethanol background noise (Fig. 5A). The strains harboring BmoR^A117T/F163Y^, BmoR^W128R^, BmoR^M94V/A117T/F163Y/F272L^ or BmoR^M94V/W128R/F272L^ exhibited negligible green fluorescence upon 100 mM ethanol exposure (Fig. 5B). Dose-response analyses revealed enhanced ethanol resistance, where BmoR^A117T/F163Y^ and BmoR^W128R^ showed reduced ethanol sensitivity compared to the wild type, while BmoR^M94V/A117T/F163Y/F272L^ and BmoR^M94V/W128R/F272L^ achieved complete ethanol insensitivity (Fig. 5C). Under simulated industrial conditions with 300 mM ethanol background noise, BmoR^M94V/A117T/F163Y/F272L^ maintained dose-dependent activation by *n*-pentanol, paralleled by the exclusive isopentanol responsiveness of BmoR^M94V/W128R/F272L^ (Fig. 5D and E). These results positioned these mutants as precision biosensors for real-time monitoring of *n*-pentanol or isopentanol in wine fermentation, overcoming traditional limitations posed by ethanol crosstalk.

### Modeling BmoR hexamer to identify key residues for pentanol orthogonality

**Fig. 6:**
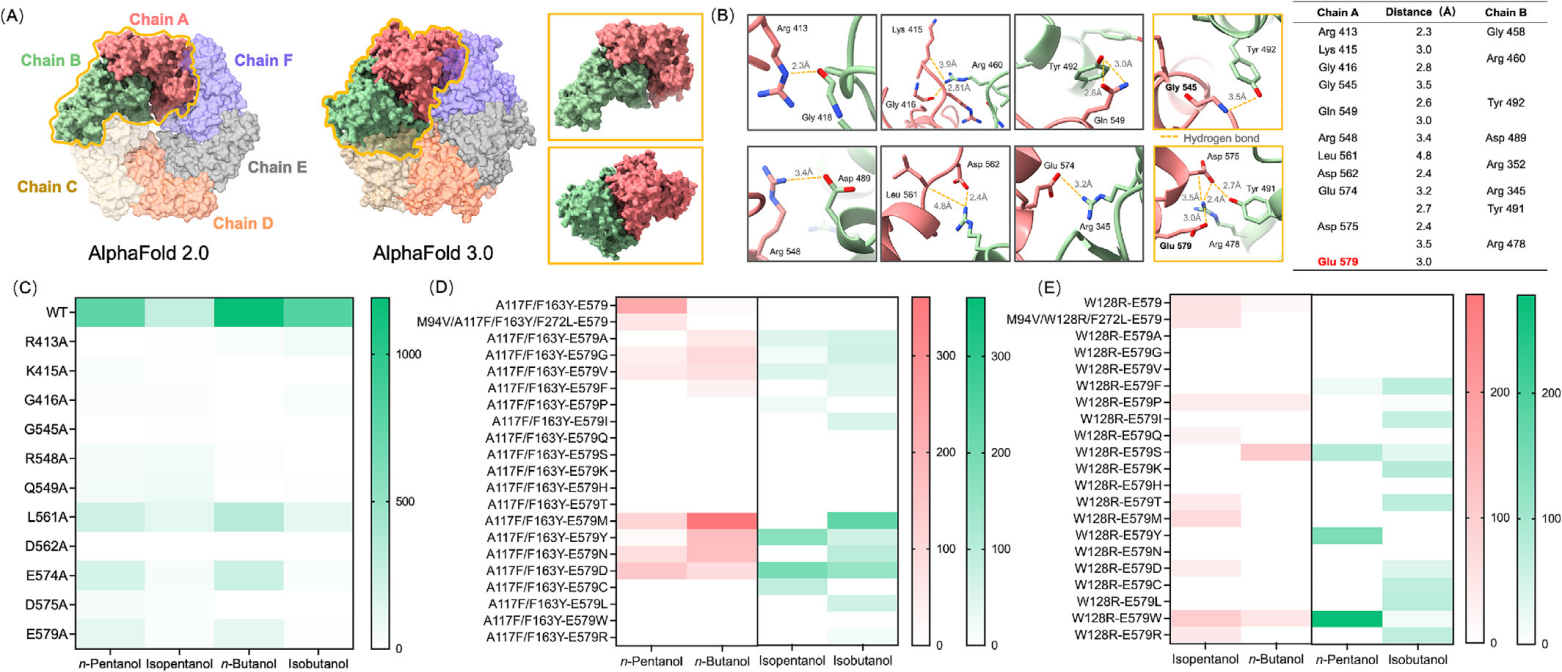
Modeling BmoR hexamer to identify key residues for pentanol orthogonality. (A) The hexamer structures of wild-type BmoR simulated by AlphaFold 2.0 and AlphaFold 3.0, respectively. (B) 11 interactions of the key residues between Chain A and Chain B. (C) The response values of alanine substitutions at 11 key residues in Chain A to 10 mM SMs. (D) The response values of BmoR^A117F/F163Y^ to four SMs after site-saturation mutagenesis at Glu579. (E) The response values of BmoR^W128R^ to four SMs after site-saturation mutagenesis at Glu579. Values and error bars represent mean and s.d. (n = 3), respectively.

To elucidate structural determinants of hexamer formation and SM orthogonality in the wild type, AlphaFold 2.0 and 3.0 structural predictions were integrated with systematic mutagenesis. Biochemical validation confirmed hexamer formation as essential for transcriptional activation at the *P_bmo_* promoter (Fig. S14). Comparative modeling revealed conserved interfacial residues across both AlphaFold predictions (Arg413, Lys415, Gly416, Gly545, Arg548, Gln549, Leu561, Asp562, Glu574, Asp575 and Glu579 in Chain A. Gly458, Arg460, Tyr492, Asp489, Arg352, Arg354, Tyr491 and Arg478 in Chain B), stabilized by eleven inter-chain HBs critical for hexamer stability (Fig. 6A and B, Table S3). Alanine scanning mutagenesis of Chain A residues further demonstrated their functional indispensability. All mutants exhibited more than 87 % reduction in GFP/OD_600_ response to 10 mM SMs compared with the wild type (Fig. 6C). Notably, Arg413A, Gly545A or Asp562A abolished *n*-pentanol sensing, while Asp562A abolished isopentanol sensing. Similarly, the Lys415A, Gly416A, Gly545A, Gln549A, Asp562A or Asp575A lost responsiveness to *n*-butanol, and the Lys415A, Gly545A, Arg548A, Asp562A or Asp575A lost responsiveness to isobutanol. Saturation mutagenesis further corroborated the centrality of these residues. All mutants showed no more than 14 % of the wild-type activity across the tested SMs (Fig. S15), which mechanistically linked the interfacial architecture to the SM discrimination capacity.

Using the hexamer of wild-type BmoR as a structural template, the hexamers of BmoR^A117T/F163Y^, BmoR^W128R^, BmoR^M94V/A117T/F163Y/F272L^ and BmoR^M94V/W128R/F272L^ were modeled to pinpoint residues governing the orthogonality (Table S4). Comparative structural analysis revealed that all four mutants acquired two additional HBs, between Ala390 in Chain A and Thr443 in Chain B, and between Arg413 in Chain A and Gly459 in Chain B, while losing a native HB between Gly545 in Chain A and Tyr492 in Chain B. Notably, BmoR^M94V/A117T/F163Y/F272L^ and BmoR^M94V/W128R/F272L^ uniquely formed an extra HB linking Glu579 in Chain A and Arg478 in Chain B. To assess the functional roles of Gly545 and Glu579, alanine substitutions were generated in the wild type. The resultant BmoR^G545A/E579A^ exhibited severely attenuated GFP/OD_600_ (Fig. S16). These findings collectively mapped critical interfacial residues that fine-tune SM discrimination through hexamer-stabilizing interactions.

The absence of a HB between Glu579 in Chain A and Arg478 in Chain B in the hexamer of BmoR^M94V/A117T/F163Y/F272L^ (versus BmoR^A117T/F163Y^) and BmoR^M94V/W128R/F272L^ (versus BmoR^W128R^) prompted the hypothesis that this interfacial interaction critically governed strict *n*-pentanol or isopentanol orthogonality. Guided by this structural insight, saturation mutagenesis on Glu579 was performed in both BmoR^A117T/F163Y^ and BmoR^W128R^. Strikingly, most Glu579 substitutions in BmoR^A117T/F163Y^ (Pro, Ile, Gln, Ser, Lys, His, Thr, Cys, Leu, Trp, Arg) and BmoR^W128R^ (Ala, Glu, Val, Phe, Lys, His, Thr, Met, Tyr, Asn, Asp, Cys, Leu, Ile, Gln, Arg) abolished *n*-butanol responsiveness (Fig. 6D and E), while the mutants of wild type Glu579 similarly exhibited attenuated *n*-butanol response (Fig. S17). These findings collectively demonstrated that strategic perturbation of Glu579 disrupted promiscuous *n*-butanol recognition, thereby enforcing SM discrimination between pentanols and their structural analogs.

### Application of strictly orthogonal mutant in the screening of isopentanol overproducers

**Fig. 7:**
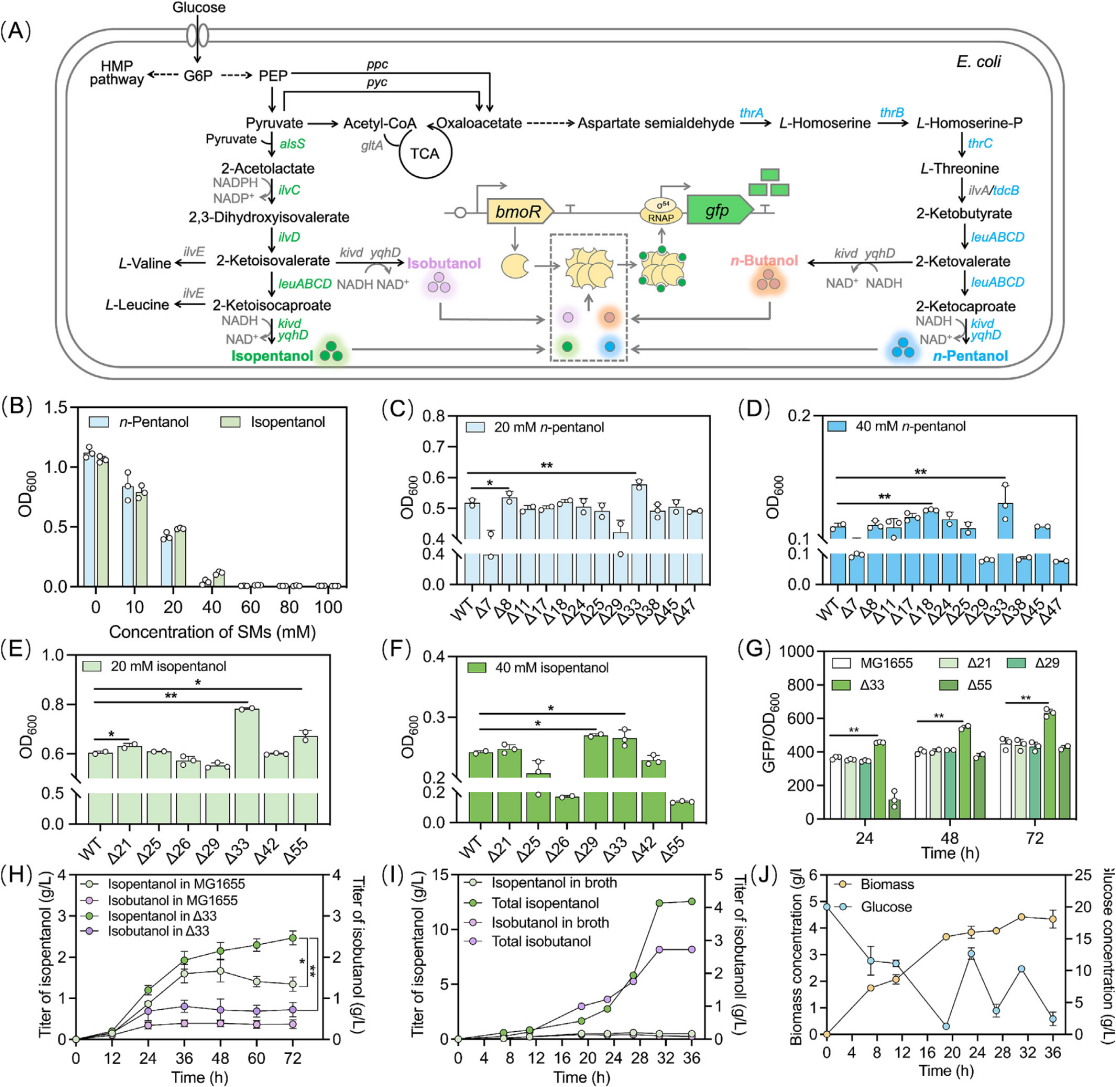
Application of strictly orthogonal mutant in isopentanol production. (A) The biosynthetic pathway of *n*-pentanol, isopentanol, *n*-butanol and isobutanol in *E. coli*. (B) The tolerance of wild-type MG1655 to *n*-pentanol and isopentanol. (C) Selection of the tolerance strains from a library of *E. coli* MG1655-derived knockout strains under the pressure of 20 mM *n*-pentanol. (D) Selection of the tolerance strain from a library of *E. coli* MG1655-derived knockout strains under the pressure of 40 mM *n*-pentanol. (E) Selection of the tolerance strain from a library of *E. coli* MG1655-derived knockout strains under the pressure of 20 mM isopentanol. (F) Selection of the tolerance strain from a library of *E. coli* MG1655-derived knockout strains under the pressure of 40 mM isopentanol. (G) Screening of the isopentanol overproducer from the pentanol-tolerant strains via BmoR^M94V/W128R/F272L^-based biosensor. (H) Shake-flask fermentation of the isopentanol overproducer. (I) The titers of isopentanol and by-product isobutanol in fed-batch fermentation. (J) The concentration of biomass and glucose in fed-batch fermentation. Values and error bars represent mean and s.d. (n = 3), respectively. *P < 0.1, ^**^P < 0.01 as determined by a two-tailed *t*-test.

To enable efficient pentanol biosynthesis (Fig. 7A), a high-throughput screen of a library of *E. coli* MG1655-derived knockout strains was initiated to enhance the tolerance of pentanols (Fig. S18). Wild-type MG1655 exhibited severe growth inhibition under the pressure of pentanols, with the OD_600_ values dropping to 0.420 ± 0.032 (2.67-fold reduction) and 0.485 ± 0.006 (2.16-fold reduction) in 20 mM *n*-pentanol and isopentanol, respectively, and failing to grow at 60 mM pentanols (Fig. 7B). Through screening, the knockout strains Δ18 and Δ33 demonstrated superior *n*-pentanol tolerance at 40 mM, achieving the OD_600_ values of 0.123 (1.33-fold increase) and 0.129 (1.29-fold increase) compared to the wild type (Fig. 7C and D). Similarly, Δ29 and Δ33 showed enhanced isopentanol resilience, registering 1.58-fold and 1.34-fold higher biomass accumulation than the wild type under equivalent isopentanol stress (Fig. 7E and F).

Leveraging the isopentanol-orthogonal BmoR^M94V/W128R/F272L^-based biosensor, an isopentanol overproducer was screened from the pre-selected isopentanol-tolerant strains (Δ21, Δ29, Δ33 and Δ55). Co-transformation of plasmids containing the expression cassettes for the biosensor and isopentanol biosynthetic pathway, into Δ33 yielded optimal performance, achieving a GFP/OD_600_ of 633 ± 23 at 24 h (Fig. 7G). Shake-flask fermentation of the engineered Δ33 demonstrated sustained isopentanol production, reaching 2.47 ± 0.17 g/L at 72 h (Fig. 7H and S19), surpassing previous benchmarks by 93 % (vs. 1.28 g/L [35]). In contrast, wild-type MG1655 plateaued at 1.60 ± 0.22 g/L by 36 h with no further accumulation. Genomic analysis of Δ33 revealed targeted knockout of 32 genes (Fig. S20 and S21), notably six genes governing amino acid transport and metabolism, and ten genes modulating energy production and conversion. In summary, using a precision-engineered isopentanol-orthogonal biosensor, the engineered Δ33 strain with a recorded isopentanol titer was efficiently screened out.

### Scale up of isopentanol production

The scaled isopentanol production of strain Δ33 was evaluated using a 3-L bioreactor (Fig. S22). Plasmids pWT-SCDA*BCD and pWT-LBD*D were con-transformed into the strain Δ33 to achieve isopentanol production. To mitigate cytotoxicity from accumulating isopentanol, the product was continuously stripped from the broth via condensation into collection bottles A and B during fermentation. During the initial 11 h post-induction, isopentanol titer reached 0.222 ± 0.016 g/L in the broth, with a cumulative titer of 0.827 g/L. Production escalated to 0.588 ± 0.029 g/L at 27 h in the broth. The glucose-to-isopentanol conversion efficiency reached 0.260 g/g, with a final titer of 12.6 g/L at 36 h (Fig. 7I), a 5.09-fold improvement over the shake-flask titer, exceeding the prior benchmark of 9.5 g/L from a mutagenized *E. coli* strain in a two-phase 60-h fermentation [36] by 33 %, while achieving this milestone in a shorter processing timeframe. Continuous glucose feeding (500 g/L stock) maintained broth concentrations above 5 g/L during the fermentation, preventing substrate limitation-induced productivity declines (Fig. 7J). The above results validated the efficacy of biosensor-driven screening strategy in identifying hyperproducers, with the strain Δ33 emerging as a premier microbial chassis for industrial-scale isopentanol biosynthesis.

## Discussion

The novelty in this study contained two-fold. First, a generalizable methodology was introduced and validated. By employing a machine learning model, the vast and intractable search space for protein mutagenesis was intelligently narrowed from hundreds of residues to a handful of critical regions, dramatically accelerating the discovery of ideal mutants. Secondly, this approach yielded engineered BmoR mutants with unprecedented SM orthogonality, capable of distinguishing between closely related structural analogs and even isomers, an achievement that was previously impractical. The practical utility of these highly specific biosensors was ultimately validated by their successful application in isolating an isopentanol overproducer, leading to a record titer. In summary, this study not only delivered a powerful new biosensing tool but also provided a blueprint for how to rationally engineer naturally flexible biological components into robust, high-precision instruments suitable for modern industrial biotechnology.

Protein ligand promiscuity, stemmed from structural congruence among substrate analogs, presents context-dependent advantages and challenges across biological systems. In enzymatic engineering, substrate promiscuity serves as a versatile platform for designing novel biosynthetic pathways, enabling both the production of target metabolites and enhanced catalytic efficiency. However, this functional flexibility introduces a critical trade-off, where unintended side reactions risk redirecting metabolic flux away from primary pathways, thereby diminishing production efficiency. TFs demonstrate analogous bifunctionality in their SM recognition capabilities. TF promiscuity allows the corresponding responsive to non-natural SMs, facilitating detection and high-throughput screening of microbial overproducers. The coexistence of structurally analogous SMs can induce cross-binding, generating false-positive signals during strain screening or enzyme evolution. This fundamental tension between versatility and orthogonality underscores the necessity for precision engineering strategies for enzymes and TFs. This work demonstrates that systematic modulation of molecular recognition domains, guided by machine learning, can resolve the SM promiscuity, enhancing precision for biosensor applications while eliminating interference from background analogs. Such tailored approaches maintain the evolutionary advantages of protein flexibility while addressing industrial demands for pathway fidelity and screening accuracy.

The SM recognition mechanism of BmoR relies predominantly on HB interactions, with BSH counts directly governing its SM selectivity. Conventional engineering approaches, such as error-prone PCR-mediated random mutagenesis [20,22], have been constrained by laborious multi-step screening processes and limited success rates. Recent advances in computational biology and machine learning have revolutionized the traditional protein engineering by enabling predictive strategies like virtual screening and molecular simulations. These methods streamline the discovery of ideal mutants through simplified pretreatment protocols and high-throughput capabilities, as exemplified by the efficient identification of bioactive compounds via sequential virtual screening [30] and the computational workflow for reprogramming TF orthogonality [37].

Building on these innovations, machine learning and molecular simulations were employed to systematically redesign BSH architecture of BmoR. The analysis revealed that modulating BSH counts directly altered SM selectivity, with the CRRs emerging as critical regions for orthogonality engineering. The machine learning model, trained on historical mutagenesis data including mutation sites T12N, M94V/F272L, and S240P from prior studies [22], pinpointed the residues in the CRRs as having large impacts on SM discrimination. Targeted CRRs mutagenesis significantly increased the likelihood of generating SM-orthogonal mutants compared to traditional random approaches. This precision strategy not only accelerated BmoR evolution but also established a generalizable framework for engineering HB-dependent TFs such as YqhC [38] and PcaV [39].

Initial high-throughput screening of the precise mutagenesis library targeting the residues within the CRRs successfully identified BmoR mutants that can distinguish *n*-pentanol from isopentanol. However, none of the mutants exhibited selectivity between *n*-butanol and pentanol isomers. To address this limitation, rational design was applied to the obtained mutants by introducing the M94V/F272L double substitution. Structural analysis revealed that these mutations disrupted a key HB interaction between Glu579 in Chain A and Arg478 in Chain B of BmoR hexamer. This modification selectively abolished the responsiveness to *n*-butanol by preventing HB formation with *n*-butanol, while preserving binding interactions with *n*-pentanol or isopentanol. Consequently, the engineered mutants achieved stringent discrimination between pentanol isomers and structural analogs, demonstrating that targeted perturbation of interchain HB networks can precisely recalibrate SM orthogonality of TFs.

In addition to BmoR^A117T/F163Y^ and BmoR^W128R^, a mutant BmoR^L209P/K254P^ was identified from the library that responded to *n*-pentanol, *n*-butanol and isobutanol but not isopentanol (Fig. S23). Subsequent attempts to eliminate butanol sensitivity in BmoR^L209P/K254P^ by introducing the mutation sites M94V/F272L resulted in a mutant BmoR^M94V/L209P/K254P/F272L^, which unexpectedly detected all four SMs (Fig. S24). Besides, previous studies demonstrated that the mutation sites W21R/E54V or I183T/D273N conferred BmoR with orthogonality for *n*-butanol over isobutanol and enabled pentanol detection [22]. To achieve strict *n*-butanol orthogonality, these mutations were introduced into BmoR^A117T/F163Y^. However, the resulting mutants BmoR^W21R/E54V/A117T/F163Y^ and BmoR^A117T/F163Y/I183T/D273N^ retained responsiveness to both pentanols and butanols (Fig. S25). Notably, combining two functional mutation sites in some mutants failed to integrate their individual response profiles, occasionally yielding opposing effects instead of dual functionalities.

The core enzymes of the isopentanol biosynthetic pathway are encoded by genes *alsS, ilvC, ilvD, leuABCD, leuDH, kivD and yqhD* [36]. Overexpression of these genes in *E. coli* MG1655 resulted in 0.852 g/L isopentanol production at 72 h (Fig. S26). To enhance isopentanol yield, LeuA was mutated to LeuA^G462D^ to alleviate feedback inhibition of 2-isopropylmalate synthase (IPMS), and KivD was modified to KivD^V461A/M538A^ to broaden its substrate binding pocket. The corresponding plasmids pWT-SCDA*BCD and pWT-LHD*D were co-transformed into *E. coli* MG1655. The engineered strain produced 0.903 ± 0.034 g/L isopentanol and 3.12 ± 0.08 g/L isobutanol at 72 h (Fig. S27) in shake-flask fermentation, indicating that the majority of carbon resources were channeled into isobutanol rather than the target product. These results highlighted the challenge of redirecting metabolic flux away from by-product formation through single-site mutagenesis of the key enzymes, emphasizing the necessity of strategic host selection with advanced screening methods.

The biosynthetic pathways of *n*-pentanol and *n*-butanol was also constructed in *E. coli* MG1655 (Fig. 7A). Overexpression of *tdcB, leuABCD, kivd* and *yqhD* did not enable the biosynthesis of *n*-pentanol or *n*-butanol. Further overexpression of *thrABC* to enhance pyruvate-to-*L*-threonine conversion still failed to produce *n*-pentanol or *n*-butanol. Notably, co-expression of plasmids pWT-SCDA*BCD and pWT-LBD*D increased isopentanol production by 1.56-fold compared to the combination of pWT-SCDA*BCD and pWT-LHD*D. The plasmid pWT-LBD*D was engineered by replacing *leuDH* with *tdcB* in the plasmid pWT-LHD*D. It was speculated that TdcB exhibited stronger *L*-leucine deamination activity as compared to LeuDH, potentially enhancing substrate processing. By optimizing the biosynthetic pathway and improving the isopentanol tolerance of the host, this study achieved the highest reported isopentanol titer to date. Nevertheless, the titer remains insufficient for industrial-scale applications, constrained by persistent limitations in the host tolerance and competition from parallel metabolic pathways that divert carbon flux away from isopentanol biosynthesis. These challenges underscore the need for further engineering to enhance both pathway specificity and microbial robustness.

Higher alcohols like pentanols and butanols contribute to the complex mouthfeel as natural by-products in wine fermentation, while excessive levels may introduce bitterness and off-flavors that compromise sensory quality [24]. *Saccharomyces cerevisiae* (*S. cerevisiae*) is a common strain in wine fermentation, but the prokaryotic σ^54^-dependent promoter *P_bmo_* restricts the application of BmoR-based biosensors in this eukaryotic host. To evaluate the potential of the biosensor for flavor compound detection, fermentation broth from *S. cerevisiae* was extracted and analyzed (Supporting Information). As illustrated in Fig. S28 and Table S5, the response values of BmoR^M94V/W128R/F272L^-based biosensor positively correlated with rising isopentanol concentrations in the fermentation broth, demonstrating its ability to detect isopentanol accurately even amid ethanol interference. Future efforts to adapt the biosensor system for *S. cerevisiae* would require engineering the *P_bmo_* promoter to operate in eukaryotic contexts, a modification that could enable real-time, *in situ* tracking of flavor compounds during the fermentation. Such advancements would address the current limitation of prokaryotic promoter dependency while expanding the utility of BmoR-biosensor in industrial fermentation processes.

## Conclusion

This study resolved the tension between protein promiscuity and industrial orthogonality by developing a machine learning-guided approach. (1) The method intelligently narrowed the mutagenesis space for BmoR, dramatically accelerating the discovery of ideal mutants. (2) These engineered BmoR mutants exhibited stringent orthogonality, discriminating between isomers and structural analogs. (3) Their practical utility was validated by successfully isolating an isopentanol overproducer that achieved a record titer of 12.6 g/L. This generalizable platform offers a new paradigm for the rational design of highly specific tools for synthetic biology and biomanufacturing.

## Data availability

The data forming the basis of this article are derived from publicly available datasets, and the sources of data have been indicated within the article. All necessary data to assess the conclusions in the paper are presented within the paper itself and/or the Supporting information.

## Compliance with ethics requirements

This manuscript does not contain any studies with human or animal subjects.

## CRediT authorship contribution statement

**Tong Wu:** Investigation, Methodology, Validation, Visualization, Writing – original draft. **Dongli Yan:** Methodology. **Sheng Lin:** Visualization. **Ran Zhang:** Investigation, Validation. **Yuhan Wang:** Investigation, Validation. **Min Li:** Investigation, Validation. **Shengzhu Yu:** Investigation. **Xiaoyan Ma:** Conceptualization. **Zhenya Chen:** Conceptualization, Funding acquisition, Project administration, Supervision, Writing – review & editing. **Yi-Xin Huo:** Conceptualization, Funding acquisition, Project administration, Supervision, Writing – review & editing.

## Declaration of competing interest

The authors declare that they have no known competing financial interests or personal relationships that could have appeared to influence the work reported in this paper.
