# Supplementary material for: Design of strictly orthogonal biosensors for maximizing renewable biofuel overproduction
Source: J Adv Res. 2025 Sep 10;84:257–72. doi: 10.1016/j.jare.2025.09.015 (PMC13227213; doi:10.1016/j.jare.2025.09.015)
Supplement: Supplementary Data 3 [file mmc3.docx]

Design of strictly orthogonal biosensors for maximizing renewable biofuel overproduction

**Supporting Information**

**Contents**

Methods

Table S1-S5

Figure S1-S28

**Methods**

**Plasmids construction**

A mutation G462D was introduced into the gene *leuA* of the plasmid pSA69-*leuABCD*, generating the plasmid pWT-SCDA^*^BCD. Two mutations V461D/M538A were introduced into the gene *kivD* of the plasmid pCS97, generating the plasmid pWT-LHD^*^D. The gene *tdcB* that amplified from the MG1655 genome replaced the gene *leuDH* of the plasmid pWT-LHD^*^D, generating the plasmid pWT-LBD^*^D. A fragment containing *P_bmoR_*-*bmoR^M94V/W128R/F272L^*-*P_bmo_*-*gfp* was amplified from the plasmid pWT-M94V/W128R/F272L, and was further inserted into the plasmid pWT-LBD^*^D, generating the plasmid pWT-B*-LBD^*^D.

A fragment containing the protein tag *sumo* was amplified from the plasmid pRSF-His-SUMO. Two fragments containing genes *bmoR* and *sfgfp* were amplified from the plasmid pYH1 and pEG1, respectively. These three fragments were assembled by OE-PCR, followed by directional cloning into the backbone containing *colE1*-*lacI-P_T7_-kan^r^* which was amplified from the plasmid pET28a-cipA-RFP-eGFP. The sequences of the TEV site and His_6_ tag were introduced via primers, finally generating the plasmid pWT-SUMO-BmoR-TEV-sfGFP-His. Subsequently, the gene *bmoR* of the plasmid pWT-SUMO-BmoR-TEV-sfGFP-His was replaced by *bmoR^M94V/A117T/F163Y/F272L^* and *bmoR^M94V/W128R/F272L^* respectively to generate the plasmids pWT-SUMO-BmoR^M94V/A117T/F163Y/F272L^-TEV-sfGFP-His and pWT-SUMO-BmoR^M94V/W128R/F272L^-TEV-sfGFP-His.

**Purification of BmoR hexamer**

Single colonies of the strain *E. coli* BL21(DE3) harboring pWT-SUMO-BmoR-TEV-sfGFP-His were inoculated into 3 mL LB with appropriate antibiotics at 37 ℃ overnight. Then 2 mL cultures were inoculated into 200 mL LB with appropriate antibiotics and cultivated at 37 ℃, 220 rpm. When the OD_600_ values reached 0.8, 0.1 mM IPTG was added into the culture and the protein was expressed at 16℃, 170 rpm for 12-18 h. After expression, the cells were collected and resuspended in binding buffer, followed by ultrasonic treatment to obtain the crude enzyme extract named Sample1. 1 mL of the crude enzyme extract was centrifuged at 13,000 rpm. The supernatant was collected named Sample2. The precipitate was resuspended using binding buffer named Sample3.

The Ni-NTA column was equilibrated with the binding buffer. The supernatant was loaded into the Ni-NTA column which was repeated 2-3 times. Then, the washing buffer containing different imidazole concentrations (20, 50, 75, 100 mM) was used to wash the column to remove non-specifically binding proteins. 200 mM elution buffer was used to elute the target protein from the column. 1mL elute was collected into 1 mL EP tube. 30 μL of the above samples were mixed with 6 µL of 6🞨protein loading buffer respectively and were boiled for 10 min. Then, the mixed samples were loaded respectively into the protein gel and run at a constant voltage of 165 V for 1 h. The protein gel was removed from the glass plates and placed into a glass box filled with water. The box was boiled for 3 min. Coomassie Brilliant Blue was used to stain the protein gel. The destaining solution was used to destain the protein gel until the bands are visible.

**Preparation and extraction of the *S. cerevisiae* fermentation broth**

Five strains of *S. cerevisiae* (M1, M2, M3, M4, M5) with different fusel oil productivities were fermented and cultured at 30 ℃ for 5 days until the weight loss was less than 0.2 g. The concentrations of ethanol and higher alcohols in fermentation liquid were measured by GC and HPLC (Table S5). The fusel oil produced by the five strains was different and there was a little difference in ethanol yield between these strains. To verify the strict orthogonality of the BmoR^M94V/W128R/F272L^-based biosensor, the fluorescence response in the fermentation solution was detected. The above fermentation solution was diluted 4 folds with LB liquid medium, then 950 μL of diluted fermentation solution was added to 96-deep wells, and then 50 μL of seed solution was added into each well.

**Code program of batch processing molecular docking**

Preparation of SM files

# python27 prepare_ligand4.py -l isobutanol.mol2

# python27 prepare_ligand4.py -l isobutanol.mol2 -A hydrogens

conda activate mko

mk_prepare_ligand -i ligand\IPA.sdf -o ligand_pdbqt\IPA.pdbqt

Preparation of BmoR mutant files

python27 prepare_receptor4.py -r receptor\E261A.pdb -o receptor_pdbqt\E261A.pdbqt -A hydrogens -U nphs

python27 prepare_receptor4.py -r receptor\E261A.pdb -o receptor_pdbqt\E261A.pdbqt -A hydrogens

Script: receptor_pdb2pdbqt.bat

## vina --config config.txt --receptor receptor_pdbqt\E261A.pdbqt --ligand ligand_pdbqt\isobutanol.pdbqt

molecular docking

vina --config config.txt --receptor receptor_pdbqt\E261A.pdbqt --ligand ligand_pdbqt\n-butanol.pdbqt --out out\E261A_n-butanol.pdbqt

Script: vina&vina_split.bat

Processing docking result（vina_split）：

vina_split --input out_ligand\E261A_n-butanol.pdbqt --ligand out_ligand_split\E261A_n-butanol

# Transform out_ligand\E261A_n-butanol.pdbqt into out_ligand_split\E261A_n-butanolx.pdbqt

Script: vina&vina_split.bat

Combining SM and BmoR, and outputting the file as pdb:

obabel receptor\E261A.pdb out_ligand_split\E261A_n-butanol1.pdbqt -opdb -O receptor_ligand_combine\E261A_n-butanol1.pdb -h

Script: receptor_ligand_combine.bat

**Table S1. Strains and plasmids used in this study**

| **Strain or plasmid** | **Genotype or description** | **Source** |
| --- | --- | --- |
| ***E. coli* strains** |  |  |
| XL10-Gold | *Tet^r^Δ(mcrA)183 Δ(mcrCB-hsdSMR-mrr)173 endA1 supE44 thi-1 recA1 gyrA96 relA1 lac Hte [F´ proAB lacI^q^ZΔM15 Tn10(Tet^r^) Amy Cam^r^]* | Stratagene |
| MG1655 | K-12; Fλ-*rph*-1 | Stratagene |
| Δ33 | MG1655 derivative, deletion of 35.976 kb fragment location (2,999,208~3,035,183) on the MG1655 genome | This study |
| **Plasmids** |  |  |
| pRSF-His-SUMO | *lacI-P_T7_-his_6_-sumo; RSF 1030; kan^r^* | Stratagene |
| pYH1 | *P_bmoR_-bmoR; P_bmo_-gfp; colE1; amp^r^* | [1] |
| pYH7 | *P_bmoR_-bmoR; P_bmo_- kan^r^; colE1; amp^r^* | [1] |
| pSA69-*leuABCD* | *P_L_lacO_1_-alsS-ilvC-ilvD-leuABCD; p15A; kan^r^* | [2] |
| pCS97 | *P_L_lacO_1_- leuDH-kivd-yqhD; colE1; amp^r^* | [3] |
| pWT-M94V/F272L | *P_bmoR_-bmoR*^M94V/F272L^*; P_bmo_-gfp; colE1; amp^r^* | [4] |
| pWT-S240P | *P_bmoR_-bmoR^S240P^; P_bmo_-gfp; colE1; amp^r^* | [4] |
| pWT-W21R/E54V | *P_bmoR_-bmoR^W21R/E54V^; P_bmo_-gfp; colE1; amp^r^* | [4] |
| pWT-I183T/D273N | *P_bmoR_-bmoR^I183T/D273N^; P_bmo_-gfp; colE1; amp^r^* | [4] |
| pEG1 | *P_bmoR_-bmoR; P_bmo_-sfgfp;* *colE1; amp^r^* | [5] |
| pET28a-cipA-RFP-eGFP | *lacI-P_T7_-cipA-rfp-egfp; colE1; amp^r^* | [6] |
| pWT-A117T/F163Y | *P_bmoR_-bmoR^A117T/F163Y^; P_bmo_-gfp; colE1; amp^r^* | This study |
| pWT-W128R | *P_bmoR_-bmoR^W128R^; P_bmo_-gfp; colE1; amp^r^* | This study |
| pWT-M94V/A117T/F163Y/F272L | *P_bmoR_-bmoR^M94V/A117T/F163Y/F272L^; P_bmo_-gfp; colE1; amp^r^* | This study |
| pWT-M94V/W128R/F272L | *P_bmoR_-bmoR^M94V/W128R/F272L^; P_bmo_-gfp; colE1; amp^r^* | This study |
| pWT-A117T/F163Y/S240P | *P_bmoR_-bmoR^A117T/F163Y/S240P^; P_bmo_-gfp; colE1; amp^r^* | This study |
| pWT-W128R/S240P | *P_bmoR_-bmoR^W128R/S240P^; P_bmo_-gfp; colE1; amp^r^* | This study |
| pWT-L209P/K254R | *P_bmoR_-bmoR^L209P/K254R^; P_bmo_-gfp; colE1; amp^r^* | This study |
| pWT-M94V/L290P/K254R/F272L | *P_bmoR_-bmoR^M94V/L290P/K254R/F272L^; P_bmo_-gfp; colE1; amp^r^* | This study |
| pWT-W21R/E54V/A117T/F163Y | *P_bmoR_-bmoR^W21R/E54V/A117T/F163Y^; P_bmo_-gfp; colE1; amp^r^* | This study |
| pWT-A117T/F163Y/I183T/D273N | *P_bmoR_-bmoR^A117T/F163Y/I183T/D273N^; P_bmo_-gfp; colE1; amp^r^* | This study |
| pWT-B^*^-LBD^*^D | *P_bmoR_-bmoR^M94V/W128R/F272L^; lacI-**P_L_lacO_1_-tdcB-kivD^V461A/M538A^-yqhD; colE1; amp^r^* | This study |
| pWT-LHD^*^D | *lacI-P_L_lacO_1_-leuDH-kivD^V461A/M538A^-yqhD; colE1; amp^r^* | This study |
| pWT-SCDA^*^BCD | *P_L_lacO_1_-alsS-ilvC-ilvD-leuA^G462D^BCD, p15A, kan^r^* | This study |
| pWT-LBD^*^D | *lacI-P_L_lacO_1_-tdcB-kivD^V461A/M538A^-yqhD; colE1; amp^r^* | This study |
| pWT-SUMO-BmoR-TEV-sfGFP-His | *lacI-P_T7_-sumo-bmoR-TEV-sfgfp-his_6_; colE1; kan^r^* | This study |
| pWT-SUMO-BmoR^M94V/A117T/F163Y/F272L^-TEV-sfGFP-His | *lacI-P_T7_-sumo-bmoR^M94V/A117T/F163Y/F272L^-TEV-sfgfp-his_6_; colE1; kan^r^* | This study |
| pWT-SUMO-BmoR^M94V/W128R/F272L^-TEV-sfGFP-His | *lacI-P_T7_-sumo-bmoR^M94V/W128R/F272L^-TEV-sfgfp-his_6_; colE1; kan^r^* | This study |

**References**

[1] H. Yu, Z. Chen, N. Wang, S. Yu, Y. Yan, Y.X. Huo. Engineering transcription factor BmoR for screening butanol overproducers. Metab Eng 2019;56: 28-38.

[2] S. Atsumi, T. Hanai, J.C. Liao. Non-fermentative pathways for synthesis of branched-chain higher alcohols as biofuels. Nature 2008;451(7174): 86-U13.

[3] Y.X. Huo, K.M. Cho, J.G.L. Rivera, E. Monte, C.R. Shen, Y.J. Yan, J.C. Liao. Conversion of proteins into biofuels by engineering nitrogen flux. Nat Biotechnol 2011;29(4): 346-U160.

[4] T. Wu, Z. Chen, S. Guo, C. Zhang, Y.X. Huo. Engineering transcription factor BmoR mutants for constructing multifunctional alcohol biosensors. ACS Synth Biol 2022;11(3): 1251-1260.

[5] M. Li, Z. Chen, W. Zhang, T. Wu, Q. Qi, Y.X. Huo. Customization of Ethylene Glycol (EG)-Induced BmoR-Based Biosensor for the Directed Evolution of PET Degrading Enzymes. Adv Sci (Weinh) 2025;12(13): e2413205.

[6] L.Y. Zhao, Z.Y. Chen, S. Lin, T. Wu, S.Z. Yu, Y.X. Huo. Biosynthesis of Isobutyraldehyde Through the Establishment of a One-Step Self-Assembly-Based Immobilization Strategy. J Agric Food Chem 2021;69(48): 14609-14619.

**Table S2.** Primers used in this study

| Primer | Sequence (5’-3’) |
| --- | --- |
| F-1 | cgacaccctgctggaagacNNNNNNNNNNNNNNNNNNNNNNNNNNNNNNNNNNNNNNNNNNNNNNNNNNNggtaccaacgctatgggtac |
| F-2 | gtccggttgctatccacggtNNNNNNNNNNNNNNNNNNNNNNNNNNNNNNNNNNNNNNNNNNNNNNNNccgatctacgacccgttcgg |
| F-3 | aggatgcagtcagcgaaggtNNNNNNNNNNNNNNNNNNNNNgatgaactgaacagccatct |
| F-4 | accgtggatagcaaccggacgacgttcagccagagcggtacccatagcgttggtacc |
| F-5 | ggacccatgtcaccagcgtaaccagagatgtccaggataccggtgaattcaccgaacgggtcgtagatcgg |
| F-6 | tacgctggtgacatgggtccggttccgatcccgttcgttcagatggctgttcagttcatc |
| F-7 | gtcttccagcagggtgtcgttaccc |
| F-8 | accttcgctgactgcatcctgctgcac |
| WT-440 | atgcatattacatacgatctgccg |
| WT-441 | ttaagcgtcaacgaaaccgg |
| WT-442 | cggcagatcgtatgtaatatgcatggtacctttctcctctttaatgaattcgg |
| WT-443 | ccggtttcgttgacgcttaagtcgacaaggagatataccatg |
| Z-9 | atgcgctggAtcaggtggatatc |
| Z-10 | tccacctgaTccagcgcatctttac |
| WT-335 | agtaacaatttgtcgagaatcggtctgatc |
| WT-455 | agagcgttcaccgacaatct |
| WT-456 | agattgtcggtgaacgctctataacgcaggaaagaacatgtgag |
| WT-457 | ccgattctcgacaaattgttactcccctgattctgtggataaccg |
| EP-001 | gtcgagaatcggtctgatccg |
| EP-002 | cggtgaaagcaccttcttcg |
| EP-003 | aagctaaacgtgctctgatc |
| EP-004 | aacagcttccaggttcagac |
| EP-005 | cgaagaaggtgctttcaccggtggtcgtc |
| EP-006 | ggatcagaccgattctcgacaaattgttac |
| E579F-F | acttcgttatgtttgttgactctggtctgcgtc |
| E579F-R | accagagtcaacaaacataacgaagtcttccg |
| E579L-F | cgttatgcttgttgactctggtctgcgtc |
| E579L-R | ccagagtcaacaagcataacgaagtcttccg |
| E579I-F | tcgttatgattgttgactctggtctgcgtc |
| E579I-R | agagtcaacaatcataacgaagtcttccgg |
| E579M-F | ttcgttatgatggttgactctggtctgcgtc |
| E579M-R | ccagagtcaaccatcataacgaagtcttccgg |
| E579V-F | ttcgttatggttgttgactctggtctgcgtc |
| E579V-R | gagtcaacaaccataacgaagtcttccg |
| E579S-F | ttcgttatgtctgttgactctggtctgcgtc |
| E579S-R | ccagagtcaacagacataacgaagtcttccg |
| E579P-F | ttcgttatgcctgttgactctggtctgcgtc |
| E579P-R | ccagagtcaacaggcataacgaagtcttccg |
| E579T-F | ttcgttatgactgttgactctggtctgcgtc |
| E579T-R | ccagagtcaacagtcataacgaagtcttccg |
| E579Y-F | ttcgttatgtatgttgactctggtctgcgtc |
| E579Y-R | gaccagagtcaacatacataacgaagtcttccg |
| E579H-F | ttcgttatgcatgttgactctggtctgcgtc |
| E579H-R | ccagagtcaacatgcataacgaagtcttccg |
| E579Q-F | ttcgttatgcaagttgactctggtctgcgtc |
| E579Q-R | ccagagtcaacttgcataacgaagtcttccg |
| E579N-F | ttcgttatgaatgttgactctggtctgcgtc |
| E579N-R | ccagagtcaacattcataacgaagtcttccg |
| E579K-F | ttcgttatgaaggttgactctggtctgcgtc |
| E579K-R | ccagagtcaaccttcataacgaagtcttccg |
| E579D-F | ttcgttatggatgttgactctggtctgcgtc |
| E579D-R | ccagagtcaacatccataacgaagtcttccg |
| E579C-F | ttcgttatgtgtgttgactctggtctgcgtc |
| E579C-R | ccagagtcaacacacataacgaagtcttccg |
| E579W-F | ttcgttatgtgggttgactctggtctgcgtcc |
| E579W-R | accagagtcaacccacataacgaagtcttccg |
| E579R-F | ttcgttatgagagttgactctggtctgcgtc |
| E579R-R | ccagagtcaactctcataacgaagtcttccg |
| E579G-F | ttcgttatgggagttgactctggtctgcgtc |
| E579G-R | ccagagtcaactcccataacgaagtcttccg |
| R413I-F | caccggtggtattcgtaaaggtaacatcgg |
| R413I-R | ctttacgaataccaccggtgaaagcacc |
| R413L-F | caccggtggtcttcgtaaaggtaacatcgg |
| R413L-R | ctttacgaagaccaccggtgaaagcacc |
| R413F-F | caccggtggttttcgtaaaggtaacatcgg |
| R413F-R | ctttacgaaaaccaccggtgaaagcacc |
| R413T-F | caccggtggtactcgtaaaggtaacatcgg |
| R413T-R | ctttacgagtaccaccggtgaaagcacc |
| R413Y-F | caccggtggttatcgtaaaggtaacatcgg |
| R413Y-R | ctttacgataaccaccggtgaaagcacc |
| R413E-F | caccggtggtgaacgtaaaggtaacatcgg |
| R413E-R | ctttacgttcaccaccggtgaaagcacc |
| R413K-F | caccggtggtaagcgtaaaggtaacatcgg |
| R413K-R | ctttacgcttaccaccggtgaaagcacc |
| R413P-F | caccggtggtcctcgtaaaggtaacatcgg |
| R413P-R | ctttacgaggaccaccggtgaaagcacc |
| R413S-F | caccggtggttctcgtaaaggtaacatcgg |
| R413S-R | ctttacgagaaccaccggtgaaagcacc |
| R413V-F | caccggtggtgttcgtaaaggtaacatcgg |
| R413V-R | ctttacgaacaccaccggtgaaagcacc |
| R413C-F | caccggtggttgtcgtaaaggtaacatcgg |
| R413C-R | ctttacgacaaccaccggtgaaagcacc |
| R413N-F | caccggtggtaatcgtaaaggtaacatcgg |
| R413N-R | ctttacgattaccaccggtgaaagcacc |
| R413D-F | caccggtggtgatcgtaaaggtaacatcgg |
| R413D-R | ctttacgatcaccaccggtgaaagcacc |
| R413G-F | caccggtggtggacgtaaaggtaacatcgg |
| R413G-R | ctttacgtccaccaccggtgaaagcacc |
| R413H-F | caccggtggtcatcgtaaaggtaacatcgg |
| R413H-R | ctttacgatgaccaccggtgaaagcacc |
| R413Q-F | caccggtggtcaacgtaaaggtaacatcgg |
| R413Q-R | ctttacgttgaccaccggtgaaagcacc |
| R413M-F | caccggtggtatgcgtaaaggtaacatcgg |
| R413M-R | ctttacgcataccaccggtgaaagcacc |
| R413W-F | caccggtggttggcgtaaaggtaacatcgg |
| R413W-R | ctttacgccaaccaccggtgaaagcacc |
| D575I-F | ccacctgccggaaattttcgttatggaagttgac |
| D575I-R | cataacgaaaatttccggcaggtggtgc |
| D575L-F | ccacctgccggaacttttcgttatggaagttgac |
| D575L-R | cataacgaaaagttccggcaggtggtgc |
| D575F-F | ccacctgccggaatttttcgttatggaagttgac |
| D575F-R | cataacgaaaaattccggcaggtggtgc |
| D575T-F | ccacctgccggaaactttcgttatggaagttgac |
| D575T-R | cataacgaaagtttccggcaggtggtgc |
| D575Y-F | ccacctgccggaatatttcgttatggaagttgac |
| D575Y-R | cataacgaaatattccggcaggtggtgc |
| D575E-F | ccacctgccggaagaattcgttatggaagttgac |
| D575E-R | cataacgaattcttccggcaggtggtgc |
| D575K-F | ccacctgccggaaaagttcgttatggaagttgac |
| D575K-R | cataacgaacttttccggcaggtggtgc |
| D575P-F | ccacctgccggaacctttcgttatggaagttgac |
| D575P-R | cataacgaaaggttccggcaggtggtgc |
| D575R-F | ccacctgccggaaagattcgttatggaagttgac |
| D575R-R | cataacgaatctttccggcaggtggtgc |
| D575S-F | ccacctgccggaatctttcgttatggaagttgac |
| D575S-R | cataacgaaagattccggcaggtggtgc |
| D575V-F | ccacctgccggaagttttcgttatggaagttgac |
| D575V-R | cataacgaaaacttccggcaggtggtgc |
| D575C-F | ccacctgccggaatgtttcgttatggaagttgac |
| D575C-R | cataacgaaacattccggcaggtggtgc |
| D575N-F | ccacctgccggaaaatttcgttatggaagttgac |
| D575N-R | cataacgaaattttccggcaggtggtgc |
| D575G-F | ccacctgccggaaggattcgttatggaagttgac |
| D575G-R | cataacgaatccttccggcaggtggtgc |
| D575H-F | ccacctgccggaacatttcgttatggaagttgac |
| D575H-R | cataacgaaatgttccggcaggtggtgc |
| D575Q-F | ccacctgccggaacaattcgttatggaagttgac |
| D575Q-R | cataacgaattgttccggcaggtggtgc |
| D575M-F | ccacctgccggaaatgttcgttatggaagttgac |
| D575M-R | cataacgaacatttccggcaggtggtgc |
| D575W-F | ccacctgccggaatggttcgttatggaagttgac |
| D575W-R | cataacgaaccattccggcaggtggtgc |
| EP-010 | accgtttcgactctgttttc |
| EP-011 | acgcagtttacggtagatggtg |
| K415G-F | ggtcgtcgtggaggtaacatcggtaaagttgctc |
| K415G-R | cgatgttacctccacgacgaccaccgg |
| K415R-F | ggtcgtcgtagaggtaacatcggtaaagttgctc |
| K415R-R | cgatgttacctctacgacgaccaccgg |
| K415W-F | ggtcgtcgttggggtaacatcggtaaagttgctc |
| K415W-R | cgatgttaccccaacgacgaccaccgg |
| K415C-F | ggtcgtcgttgtggtaacatcggtaaagttgctc |
| K415C-R | cgatgttaccacaacgacgaccaccgg |
| K415E-F | ggtcgtcgtgaaggtaacatcggtaaagttgctc |
| K415E-R | cgatgttaccttcacgacgaccaccgg |
| K415D-F | ggtcgtcgtgatggtaacatcggtaaagttgctc |
| K415D-R | cgatgttaccatcacgacgaccaccgg |
| K415N-F | ggtcgtcgtaatggtaacatcggtaaagttgctc |
| K415N-R | cgatgttaccattacgacgaccaccgg |
| K415Q-F | ggtcgtcgtcaaggtaacatcggtaaagttgctc |
| K415Q-R | cgatgttaccttgacgacgaccaccgg |
| K415H-F | ggtcgtcgtcatggtaacatcggtaaagttgctc |
| K415H-R | cgatgttaccatgacgacgaccaccgg |
| K415Y-F | ggtcgtcgttatggtaacatcggtaaagttgctc |
| K415Y-R | cgatgttaccataacgacgaccaccgg |
| K415A-F | ggtcgtcgtgctggtaacatcggtaaagttgctc |
| K415A-R | cgatgttaccagcacgacgaccaccgg |
| K415T-F | ggtcgtcgtactggtaacatcggtaaagttgctc |
| K415T-R | cgatgttaccagtacgacgaccaccgg |
| K415P-F | ggtcgtcgtcctggtaacatcggtaaagttgctc |
| K415P-R | cgatgttaccaggacgacgaccaccgg |
| K415S-F | ggtcgtcgttctggtaacatcggtaaagttgctc |
| K415S-R | cgatgttaccagaacgacgaccaccgg |
| K415V-F | ggtcgtcgtgttggtaacatcggtaaagttgctc |
| K415V-R | cgatgttaccaacacgacgaccaccgg |
| K415M-F | ggtcgtcgtatgggtaacatcggtaaagttgctc |
| K415M-R | cgatgttacccatacgacgaccaccgg |
| K415I-F | ggtcgtcgtattggtaacatcggtaaagttgctc |
| K415I-R | cgatgttaccaatacgacgaccaccgg |
| K415L-F | ggtcgtcgtcttggtaacatcggtaaagttgctc |
| K415L-R | cgatgttaccaagacgacgaccaccgg |
| K415F-F | ggtcgtcgttttggtaacatcggtaaagttgctc |
| K415F-R | cgatgttaccaaaacgacgaccaccgg |
| c-545-549-548-F | gaccctgttcaaacgtcacgcttg |
| c-545-549-548-R | cagcgtccagaacgttgtgcag |
| c-575-579-574-F | gtcacgttatcgaaccgcaccac |
| c-575-579-574-R | gatcggacgcagaccagagtcaac |
| Q549I-F | taacctgcgtattctgcacaacgttctgg |
| Q549I-R | cgttgtgcagaatacgcaggttacccg |
| Q549L-F | taacctgcgtcttctgcacaacgttctgg |
| Q549L-R | cgttgtgcagaagacgcaggttacccg |
| Q549F-F | taacctgcgttttctgcacaacgttctgg |
| Q549F-R | cgttgtgcagaaaacgcaggttacccg |
| Q549T-F | taacctgcgtactctgcacaacgttctgg |
| Q549T-R | cgttgtgcagagtacgcaggttacccg |
| Q549Y-F | taacctgcgttatctgcacaacgttctgg |
| Q549Y-R | cgttgtgcagataacgcaggttacccg |
| Q549E-F | taacctgcgtgaactgcacaacgttctgg |
| Q549E-R | cgttgtgcagttcacgcaggttacccg |
| Q549K-F | taacctgcgtaagctgcacaacgttctgg |
| Q549K-R | cgttgtgcagcttacgcaggttacccg |
| Q549P-F | taacctgcgtcctctgcacaacgttctgg |
| Q549P-R | cgttgtgcagaggacgcaggttacccg |
| Q549R-F | taacctgcgtagactgcacaacgttctgg |
| Q549R-R | cgttgtgcagtctacgcaggttacccg |
| Q549S-F | taacctgcgttctctgcacaacgttctgg |
| Q549S-R | cgttgtgcagagaacgcaggttacccg |
| Q549V-F | taacctgcgtgttctgcacaacgttctgg |
| Q549V-R | cgttgtgcagaacacgcaggttacccg |
| Q549C-F | taacctgcgttgtctgcacaacgttctgg |
| Q549C-R | cgttgtgcagacaacgcaggttacccg |
| Q549N-F | taacctgcgtaatctgcacaacgttctgg |
| Q549N-R | cgttgtgcagattacgcaggttacccg |
| Q549D-F | taacctgcgtgatctgcacaacgttctgg |
| Q549D-R | cgttgtgcagatcacgcaggttacccg |
| Q549G-F | taacctgcgtggactgcacaacgttctgg |
| Q549G-R | cgttgtgcagtccacgcaggttacccg |
| Q549H-F | taacctgcgtcatctgcacaacgttctgg |
| Q549H-R | cgttgtgcagatgacgcaggttacccg |
| Q549M-F | taacctgcgtatgctgcacaacgttctgg |
| Q549M-R | cgttgtgcagcatacgcaggttacccg |
| Q549W-F | taacctgcgttggctgcacaacgttctgg |
| Q549W-R | cgttgtgcagccaacgcaggttacccg |
| 413-F | tcaccggtggtgctcgtaaa |
| 413-R | ttacgagcaccaccggtgaaagcac |
| 460-F | tggtgctgaaccgatgccggttgacata |
| 460-R | cggcatcggttcagcacca |
| 416-F | aaagctaacatcggtaaagttgctcaggc |
| 416-R | ctttaccgatgttagctttacgacgac |
| 574-F | gccggctgacttcgttatggaag |
| 574-R | cataacgaagtcagccggcaggtg |
| 561-F | ggctatggctgacgacggtcacgttatc |
| 561-R | accgtcgtcagccatagccagag |
| 562-F | atgctggctgacggtcacgttatc |
| 562-R | acgtgaccgtcagccagcatagc |
| 575-F | gccggaagctttcgttatggaagttg |
| 575-R | cataacgaaagcttccggcaggtggtg |
| 579-F | atggctgttgactctggtctgc |
| 579-R | gaccagagtcaacagccataacgaag |
| 545-F | aaacgtcacgcttggccggctaac |
| 545-R | caggttagccggccaagcgtga |
| 549-F | cctgcgtgctctgcacaacgttc |
| 549-R | gttgtgcagagcacgcaggttaccc |
| 548-F | aacctggctcagctgcacaacgttc |
| 548-R | tgtgcagctgagccaggttacc |
| 545-Phe-F | acgcttggccgtttaacctgc |
| 545-Phe-R | aggttaaacggccaagcgtgac |
| 545-Leu-F | tcacgcttggccgcttaacctgc |
| 545-Leu-R | gttaagcggccaagcgtgacgtttg |
| 545-Ile-F | acgcttggccgattaacctgc |
| 545-Ile-R | ggttaatcggccaagcgtgac |
| 545-Met-F | gccgatgaacctgcgtcagctgca |
| 545-Met-R | ctgacgcaggttcatcggccaagc |
| 545-Ser-F | tcacgcttggccgtctaacctgc |
| 545-Ser-R | gttagacggccaagcgtgacgtttg |
| 545-Pro-F | ttggccgcctaacctgcgtcagctgca |
| 545-Pro-R | acgcaggttaggcggccaagc |
| 545-Thr-F | cgcttggccgactaacctgcgtc |
| 545-Thr-R | gcaggttagtcggccaagcgtgac |
| 545-Val-F | acgcttggccggttaacctg |
| 545-Val-R | aggttaaccggccaagcgtgac |
| 545-Tyr-F | cacgcttggccgtataacctgc |
| 545-Tyr-R | ggttatacggccaagcgtgac |
| 545-His-F | acgcttggccgcataacctgc |
| 545-His-R | acgcaggttatgcggccaagc |
| 545-Gln-F | cgcttggccgcaaaacctgcgtc |
| 545-Gln-R | caggttttgcggccaagcgtgac |
| 545-Asn-F | cttggccgaataacctgcgtc |
| 545-Asn-R | cgcaggttattcggccaagc |
| 545-Lys-F | gtcacgcttggccgaagaacctgcgtc |
| 545-Lys-R | aggttcttcggccaagcgtgac |
| 545-Asp-F | ttggccggataacctgcgtc |
| 545-Asp-R | tgacgcaggttatccggcca |
| 545-Glu-F | ttggccggaaaacctgcgtc |
| 545-Glu-R | tgacgcaggttttccggcca |
| 545-Cys-F | acgcttggccgtgtaacctg |
| 545-Cys-R | aggttacacggccaagcgtg |
| 545-Trp-F | tcacgcttggccgtggaacctgcgtc |
| 545-Trp-R | tgacgcaggttccacggccaagc |
| 545-Arg-F | gcttggccgagaaacctgcgtc |
| 545-Arg-R | gcaggtttctcggccaagcgtga |
| 548-Phe-F | aacctgtttcagctgcacaacgttc |
| 548-Phe-R | tgtgcagctgaaacaggttacccgg |
| 548-Leu-F | aacctgcttcagctgcacaacgttc |
| 548-Leu-R | tgtgcagctgaagcaggttacccg |
| 548-Ile-F | cctgattcagctgcacaacg |
| 548-Ile-R | gttgtgcagctgaatcaggttac |
| 548-Met-F | cctgatgcagctgcacaacgttc |
| 548-Met-R | aacgttgtgcagctgcatcaggttaccc |
| 548-Val-F | gtaacctggttcagctgcaca |
| 548-Val-R | tgcagctgaaccaggttaccc |
| 548-Ser-F | ggtaacctgtctcagctgcacaacgt |
| 548-Ser-R | ttgtgcagctgagacaggttacccgg |
| 548-Pro-F | ggtaacctgcctcagctgcacaac |
| 548-Pro-R | ttgtgcagctgaggcaggttaccc |
| 548-Thr-F | gtaacctgactcagctgcacaac |
| 548-Thr-R | tgcagctgagtcaggttacc |
| 548-Tyr-F | cgggtaacctgtatcagctgcac |
| 548-Tyr-R | gcagctgatacaggttacccgg |
| 548-His-F | gggtaacctgcatcagctgcac |
| 548-His-R | cagctgatgcaggttacccg |
| 548-Gln-F | gtaacctgcaacagctgcacaacgt |
| 548-Gln-R | tgcagctgttgcaggttacccgg |
| 548-Asn-F | gtaacctgaatcagctgcacaacgttctg |
| 548-Asn-R | ttgtgcagctgattcaggttacccgg |
| 548-Lys-F | ccgggtaacctgaagcagctgcacaac |
| 548-Lys-R | gctgcttcaggttacccggccaa |
| 548-Asp-F | gtaacctggatcagctgcacaac |
| 548-Asp-R | tgcagctgatccaggttacc |
| 548-Glu-F | gtaacctggaacagctgcacaac |
| 548-Glu-R | tgcagctgttccaggttaccc |
| 548-Cys-F | gggtaacctgtgtcagctgcacaac |
| 548-Cys-R | cagctgacacaggttacccgg |
| 548-Trp-F | aacctgtggcagctgcacaac |
| 548-Trp-R | tgtgcagctgccacaggttac |
| 548-Gly-F | ggtaacctgggacagctgcacaacgtt |
| 548-Gly-R | ttgtgcagctgtcccaggttacccg |
| Kivd-461-F | acagccgaaagagaaattcatggaccaaatc |
| Kivd-461-R | ggtccatgaatttctctttcggctgtataaccatc |
| Kivd-538-F | ctgaaaaaagcgggcaaactatttgctgaac |
| Kivd-538-R | atagtttgcccgcttttttcagtacttttggtgcatcttc |
| LeuA-462-F | atgcgctggatcaggtggatatc |
| LeuA-462-R | tccacctgatccagcgcatctttac |

**Table S3.** The interactions between Chain A and Chain B in the hexamer of wild-type BmoR by AlphaFold 3.0

| Chain A | Distance (Å) | Chain B |
| --- | --- | --- |
| Gln5 | 3.2 | Lys3 |
| Asp33 | 3.0 | Arg119 |
| Arg146 | 2.1 | Glu115 |
| Gln199 | 3.4 | Asp111 |
| Arg225 | 3.0 | Glu86 |
| Arg284 | 3.0 | His425 |
| Arg413 | 2.6 | Glu405 |
|  | 3.0 |  |
|  | 2.3 | Gly458 |
| Thr410 | 2.5 | Thr410 |
| Asn417 | 3.1 | Arg460 |
|  | 3.5 |  |
| Ala390 | 2.4 | Thr443 |
| Asp436 | 2.6 | Arg487 |
|  | 3.3 |  |
| Glu367 | 2.9 | Arg447 |
|  | 2.8 |  |
| Arg548 | 2.8 | Asp489 |
| Asn552 | 2.0 | Tyr492 |
|  | 3.5 | Asn495 |
| Asp575 | 2.6 |  |
| Glu579 | 3.1 | Arg478 |
|  | 2.6 |  |
| Glu574 | 2.5 | Arg345 |
|  | 3.2 |  |
|  | 3.4 | Arg641  Arg641 |
| Pro573 | 2.8 |  |
| Leu572 | 2.8 |  |
| Pro569 | 3.4 |  |
| Asp555 | 3.2 | Arg352 |
| Asp562 | 2.4 | Arg348 |
|  | 3.1 |  |
| Met560 | 3.1 |  |
| Glu568 | 2.7 | Arg649 |
| Asp581 | 2.6 | Ser631 |
|  | 3.1 |  |

**Table S4.** The interactions between Chain A and Chain B in the hexamer of BmoR mutants

| Chain A | Chain B | BmoR mutants |
| --- | --- | --- |
| Arg413 | Gly458 | - WT - A117T/F163Y - W128R - M94V/A117T/F163Y/F272L - M94V/W128R/F272L |
|  | Gly459 | - A117T/F163Y - W128R - M94V/A117T/F163Y/F272L - M94V/W128R/F272L |
| Lys415 | Arg460 | - WT - A117T/F163Y - W128R - M94V/A117T/F163Y/F272L - M94V/W128R/F272L |
| Gly416 |  | - WT - A117T/F163Y - W128R - M94V/A117T/F163Y/F272L - M94V/W128R/F272L |
| Gly545 | Tyr492 | - WT |
| Gln549 |  | - WT - A117T/F163Y - W128R - M94V/A117T/F163Y/F272L - M94V/W128R/F272L |
| Arg548 | Asp489 | - WT - A117T/F163Y - W128R - M94V/A117T/F163Y/F272L - M94V/W128R/F272L |
| Leu561 | Arg352 | - WT - A117T/F163Y - W128R - M94V/A117T/F163Y/F272L - M94V/W128R/F272L |
| Asp 562 |  | - WT - A117T/F163Y - W128R - M94V/A117T/F163Y/F272L - M94V/W128R/F272L |
| Glu574 | Arg345 | - WT - A117T/F163Y - W128R - M94V/A117T/F163Y/F272L - M94V/W128R/F272L |
| Asp575 | Tyr491 | - WT - A117T/F163Y - W128R - M94V/A117T/F163Y/F272L - M94V/W128R/F272L |
|  | Arg478 | - WT - A117T/F163Y - W128R - M94V/A117T/F163Y/F272L - M94V/W128R/F272L |
|  |  | - WT - A117T/F163Y - W128R |
| Glu579 |  |  |
| Ala390 | Thr443 | - A117T/F163Y - W128R - M94V/A117T/F163Y/F272L - M94V/W128R/F272L |

**Table S5.** Contents of higher alcohols and ethanol

| Strains | *n*-Propanol（mg/L） | Isobutanol（mg/L） | Isopentanol（mg/L） | Ethanol（g/L） | The total higher alcohols（mg/L） |
| --- | --- | --- | --- | --- | --- |
| M1 | 61.77 | 34.74 | 249.03 | 105.3 | 400.12 |
| M2 | 64.19 | 102.24 | 451.64 | 123.2 | 640.92 |
| M3 | 110.71 | 133.59 | 656.08 | 117.3 | 961.23 |
| M4 | 81.86 | 393.95 | 585.07 | 113.1 | 1109.75 |
| M5 | 107.59 | 464.95 | 720.24 | 117.13 | 1317.42 |


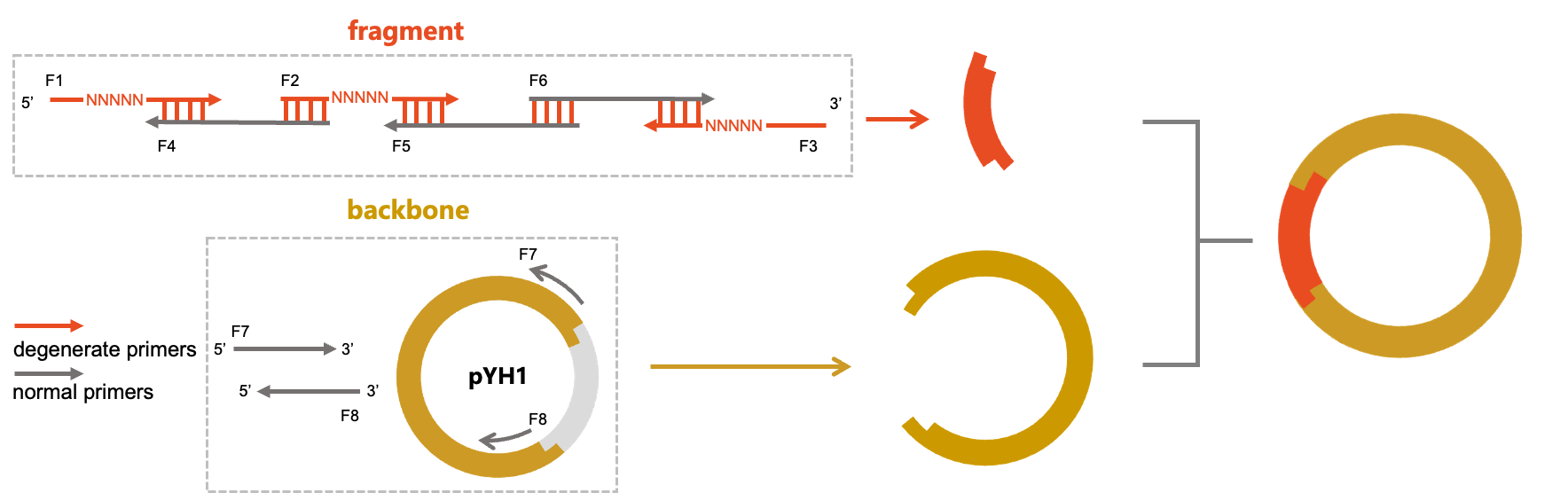


**Fig. S1 Establishment of the precise mutagenesis library.** A fragment covering the residues within the CRRs was synthesized using degenerate primers and normal primers. A backbone was amplified using the plasmid pYH1 as the template. The fragment and the backbone were assembled and transferred into *E. coli*. A precise mutagenesis library targeting the residues within the CRRs was established.


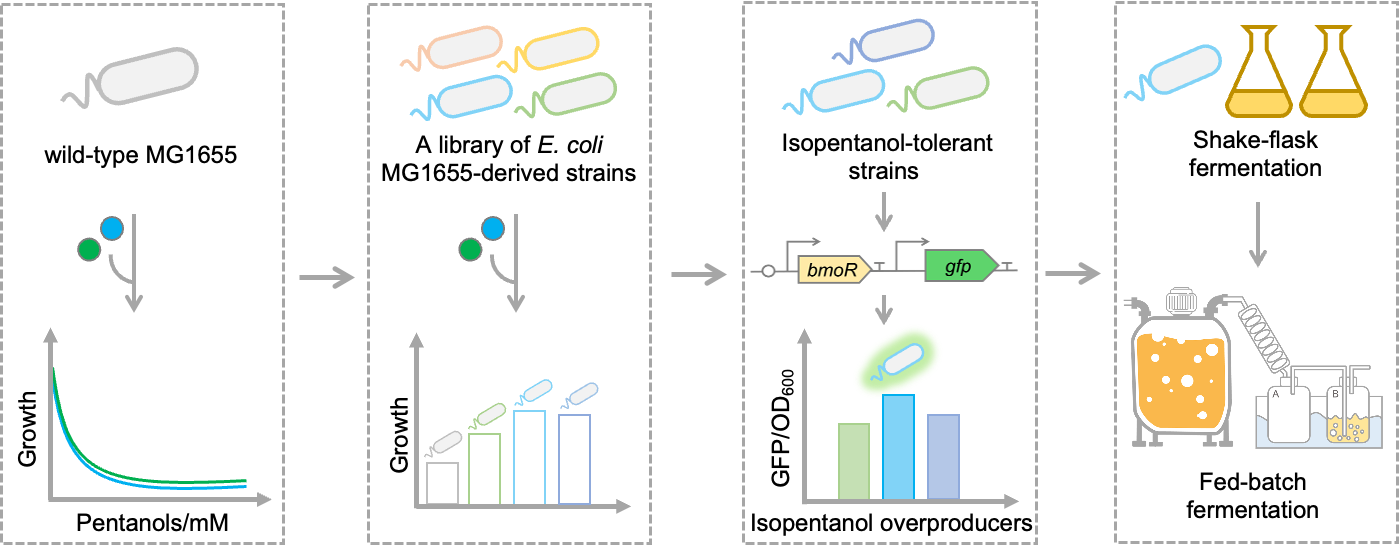


**Fig. S2** **The whole screening process for pentanol-tolerant strains and overproducers.** Firstly, the tolerance of wild-type MG1655 towards *n*-pentanol and isopentanol was measured respectively. Then, pentanol-tolerant strains were screened out from a library of *E. coli* MG1655-derived strains in the pressure of 20 or 40 mM pentanols. Next, an isopentanol overproducer was screened out from the pentanol-tolerant strains using BmoR^M94V/W128R/F272L^-based biosensor. Finally, the isopentanol production of the isopentanol overproducer was verified by shake-flask fermentation and fed-batch fermentation.

**
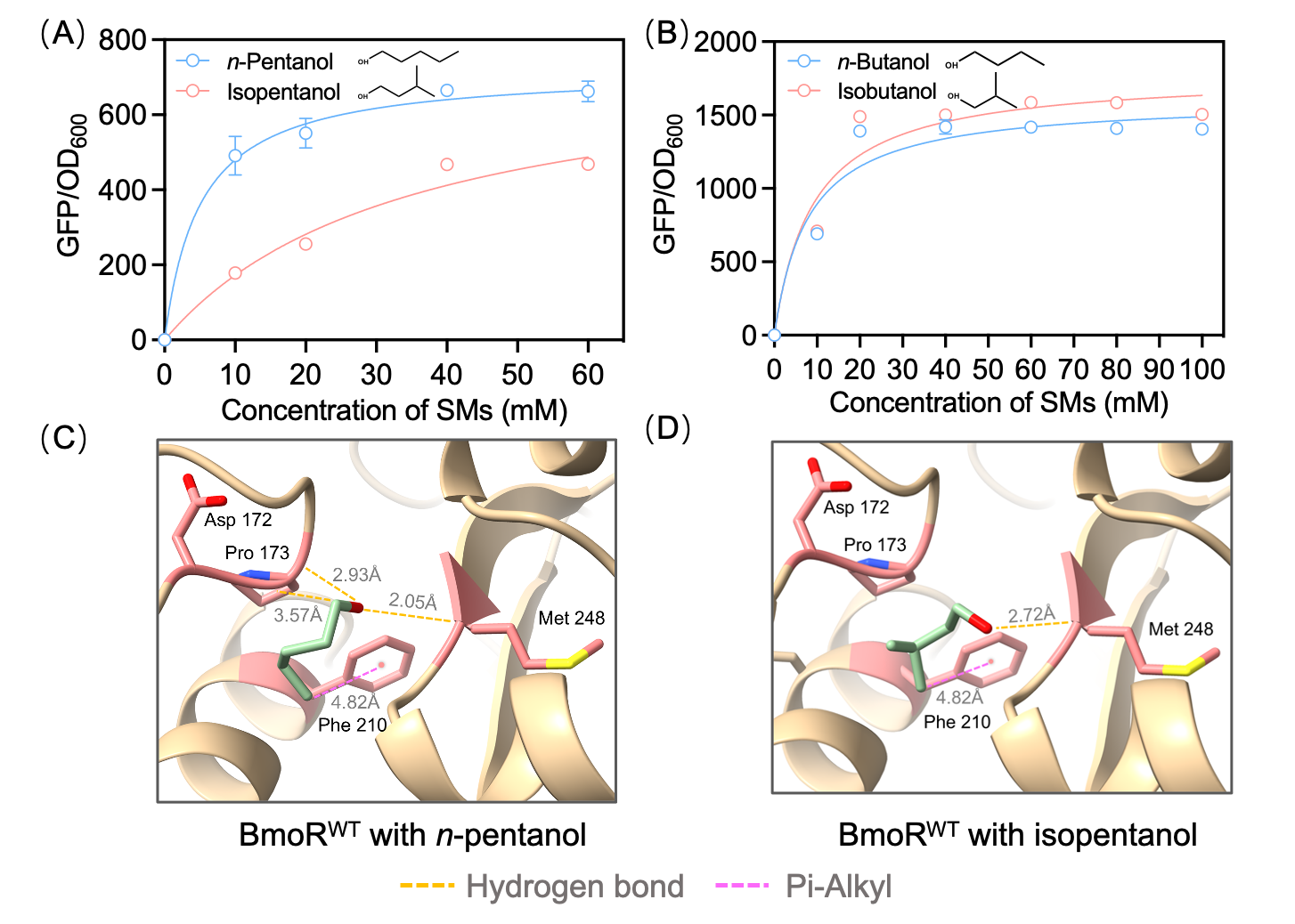
**

**Fig. S3 The response values of** **wild-type BmoR-based biosensor to** ***n*-pentanol, isopentanol, *n*-butanol or isobutanol and molecular simulations of BmoR with SMs.** (A) Response curves of wild-type BmoR towards *n*-pentanol or isopentanol. (B) Response curves of wild-type BmoR towards *n*-butanol or isobutanol. (C) Molecular simulations of wild-type BmoR with *n*-pentanol. (D) Molecular simulations of wild-type BmoR with isopentanol. Values and error bars represent mean and s.d. (n = 3), respectively.

**
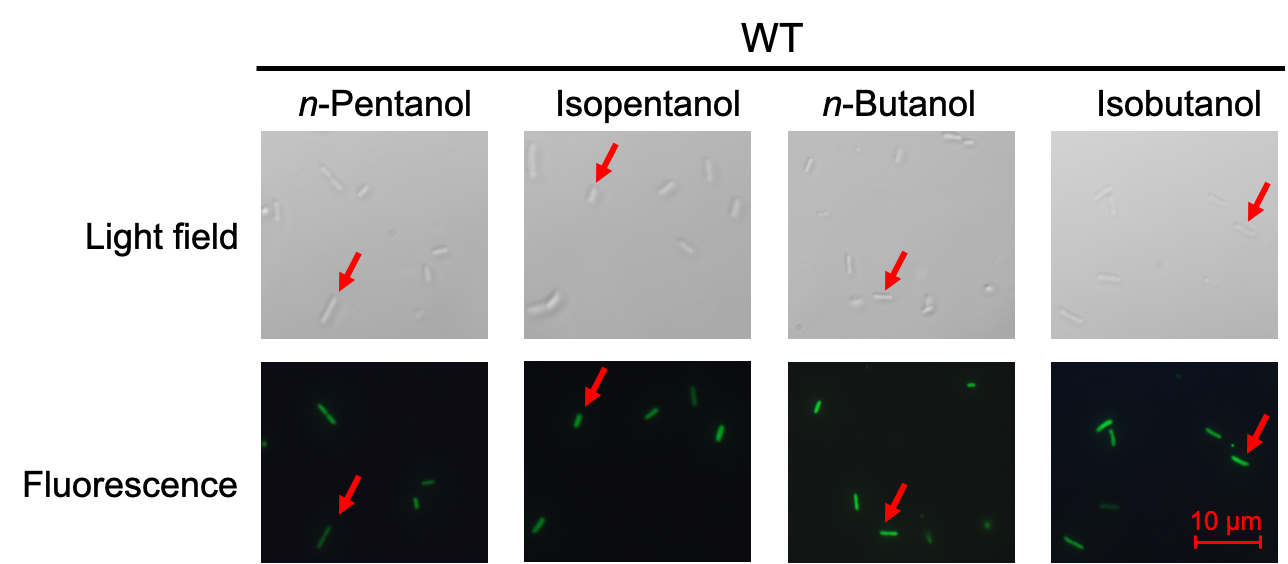
**

**Fig. S4 Green fluorescence of the strains harboring wild-type BmoR in presence of 10 mM *n*-pentanol, isopentanol, *n*-butanol or isobutanol.**

**
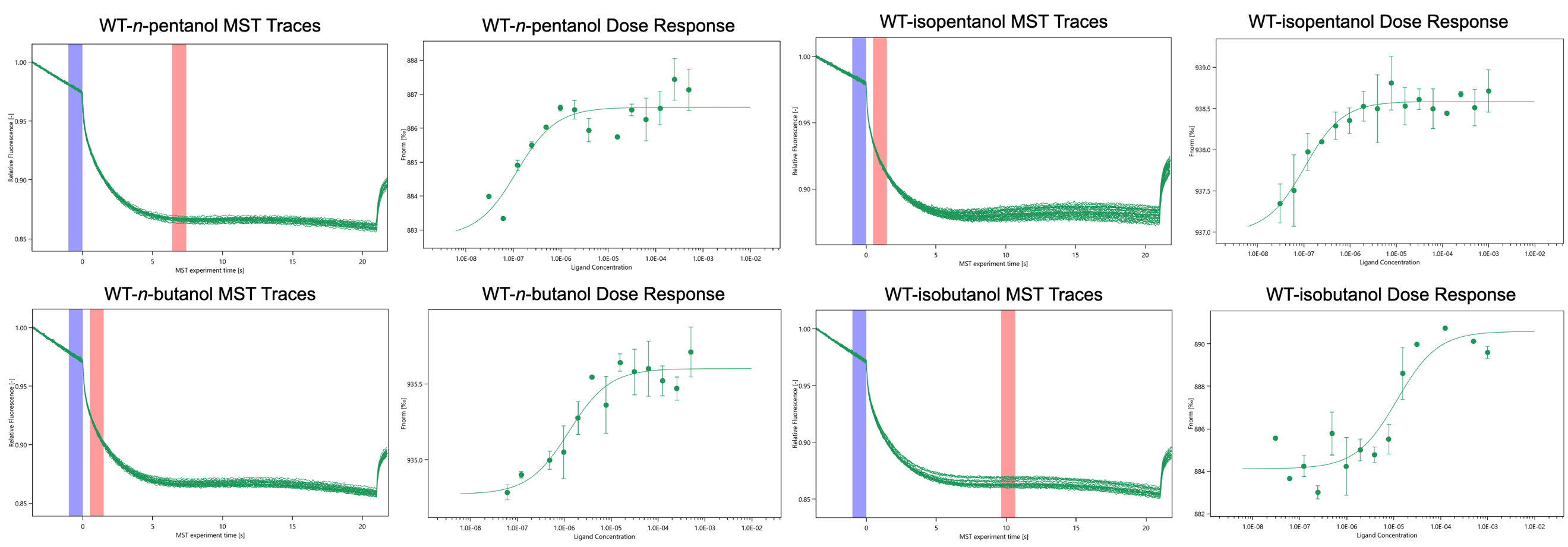
**

**Fig. S5 The fnorm fitting graphs of wild-type BmoR towards *n*-pentanol, isopentanol, *n*-butanol or isobutanol.**

**
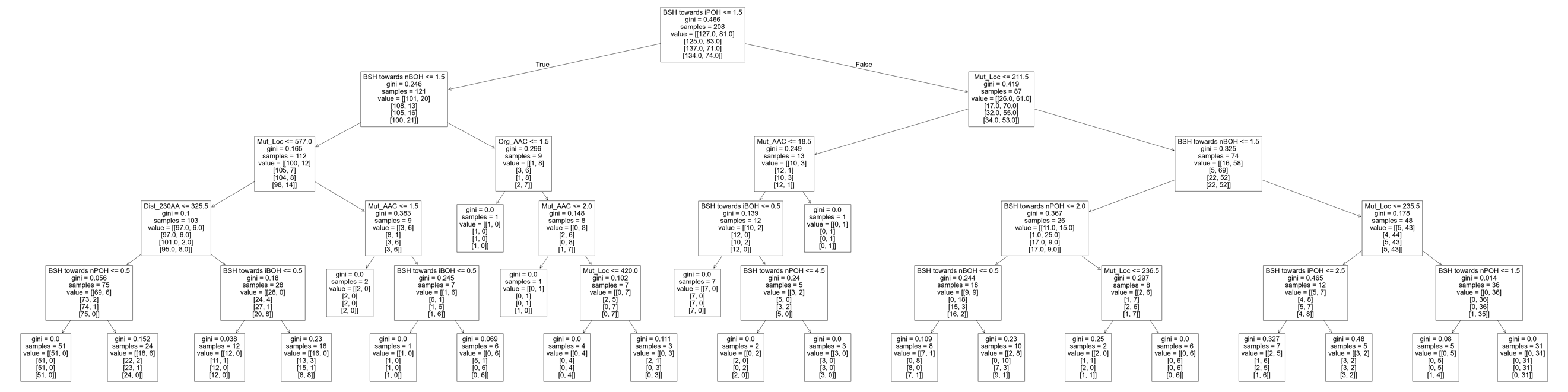
**

**Fig. S6** **Random Forest Algorithm to train on the learning dataset.**

**
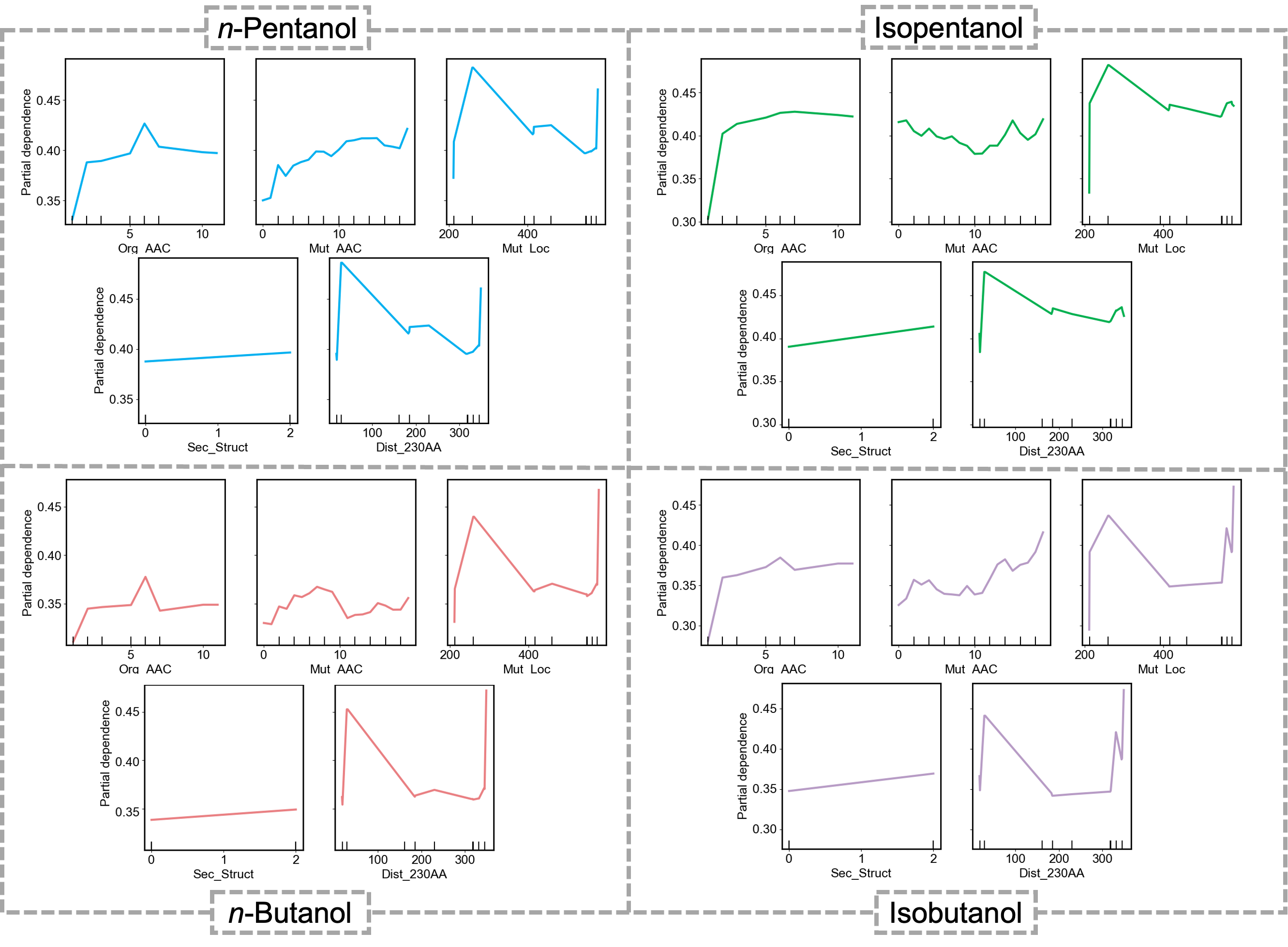
**

**Fig. S7 The corresponding partial dependence plots of input parameters.** The input parameters included residue characteristics pre-/post-mutation, mutation site-containing domain, mutation site-embedded secondary domain and mutation site-to-SBD distance. These plots quantified the marginal effect of these four parameters on the response of BmoR toward four SMs. Org_AAC: residue characteristics pre-mutation. Mut_AAC: residue characteristics post-mutation. Struct_dom: mutation site-containing domain. Sec_Struct: mutation site-embedded secondary domain. Dis_230AA: mutation site-to-SBD distance.

**
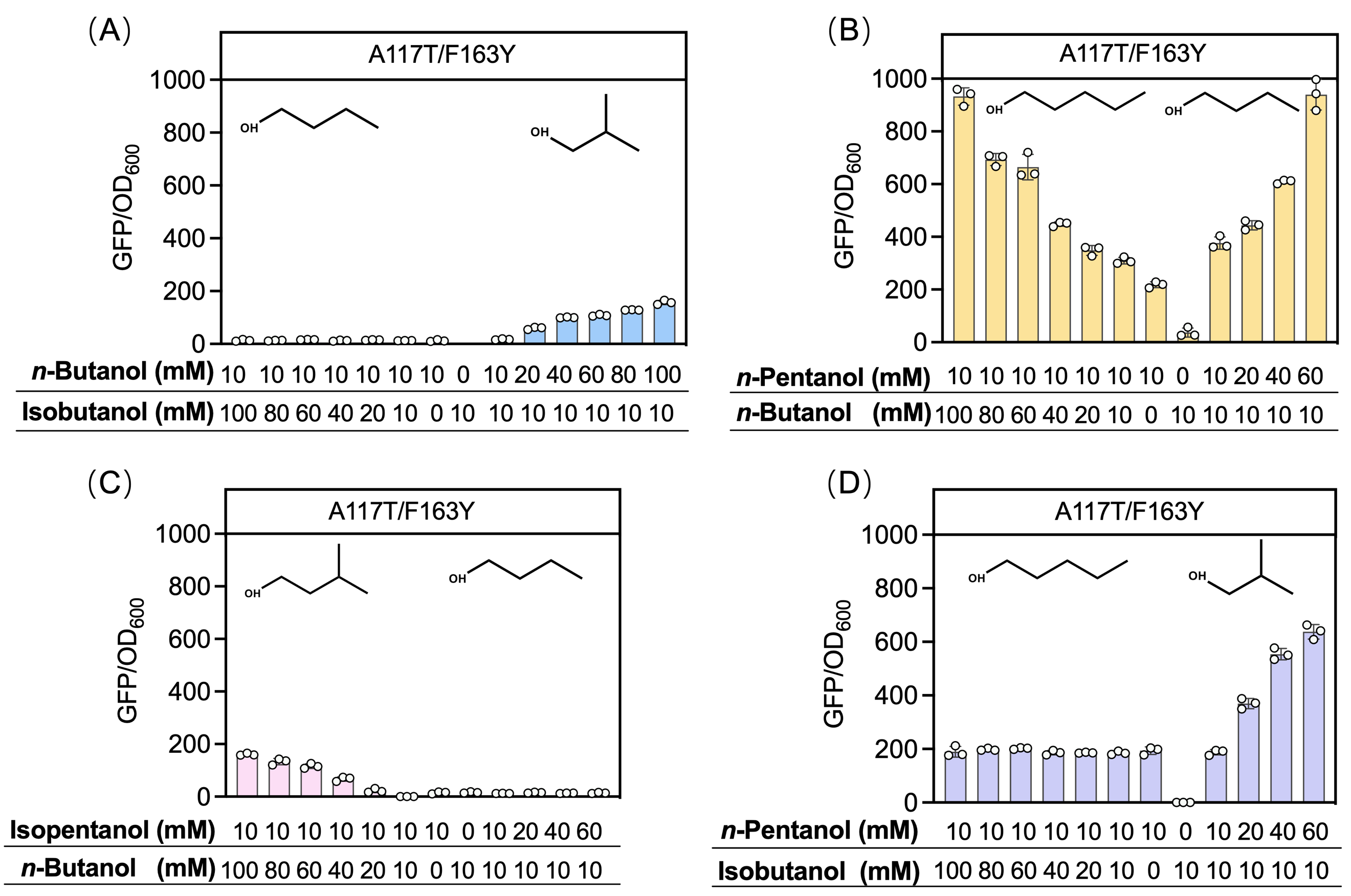
**

**Fig. S8** **The response values of BmoR^A117T/F163Y^-based biosensor to *n*-pentanol, isopentanol, *n*-butanol or isobutanol.** (A) Maintaining the concentration (10 mM) of *n*-butanol or isobutanol and increasing the concentration of *n*-butanol or isobutanol with a gradient (0-100 mM) to confirm the orthogonality of BmoR^A117F/F163Y^ towards *n*-pentanol and *n*-butanol. (B) Maintaining the concentration (10 mM) of *n*-pentanol or *n*-butanol and increasing the concentration of *n*-pentanol or *n*-butanol with a gradient (0-100 mM or 0-60 mM) to confirm the orthogonalityof BmoR^A117F/F163Y^ towards *n*-pentanol and *n*-butanol. (C) Maintaining the concentration (10 mM) of isopentanol or *n*-butanol and increasing the concentration of isopentanol or *n*-butanol with a gradient (0-60 mM or 0-100 mM) to confirm the orthogonality of BmoR^A117F/F163Y^ towards *n*-pentanol and *n*-butanol. (D) Maintaining the concentration (10 mM) of *n*-pentanol or isobutanol and increasing the concentration of *n*-pentanol or isobutanol with a gradient (0-60 mM or 0-100 mM) to confirm the orthogonality of BmoR^A117F/F163Y^ towards *n*-pentanol and *n*-butanol. Values and error bars represent mean and s.d. (n = 3), respectively.

**
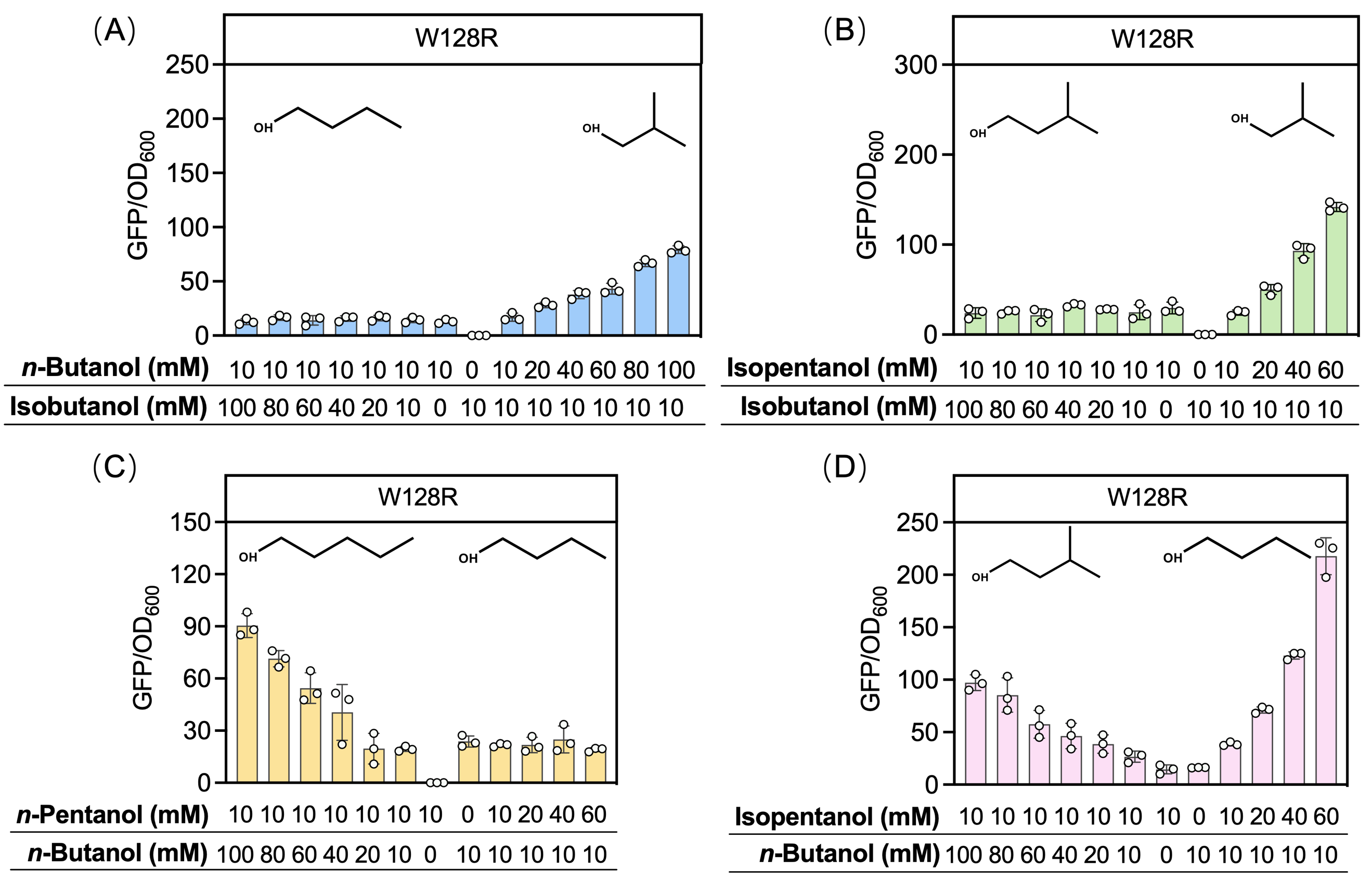
**

**Fig. S9 The response values of BmoR^W128R^-based biosensor to *n*-pentanol, isopentanol, *n*-butanol or isobutanol.** (A) Maintaining the concentration (10 mM) of *n*-butanol or isobutanol and increasing the concentration of *n*-butanol or isobutanol with a gradient (0-100 mM) to confirm the orthogonality of BmoR^W128R^ towards isopentanol and *n*-butanol. (B) Maintaining the concentration (10 mM) of isopentanol or isobutanol, and increasing the concentration of isopentanol or isobutanol with a gradient (0-60 mM or 0-100 mM) to confirm the orthogonalityof BmoR^W128R^ towards isopentanol and *n*-butanol.(C) Maintaining the concentration (10 mM) of *n*-pentanol or *n*-butanol, and increasing the concentration of *n*-pentanol or *n*-butanol with a gradient (0-60 mM or 0-100 mM) to confirm the orthogonality of BmoR^W128R^ towards isopentanol and *n*-butanol. (D) Maintaining the concentration (10 mM) of isopentanol or *n*-butanol, and increasing the concentration of isopentanol or *n*-butanol with a gradient (0-60 mM or 0-100 mM) to confirm the orthogonality of BmoR^W128R^ towards isopentanol and *n*-butanol. Values and error bars represent mean and s.d. (n = 3), respectively.

**
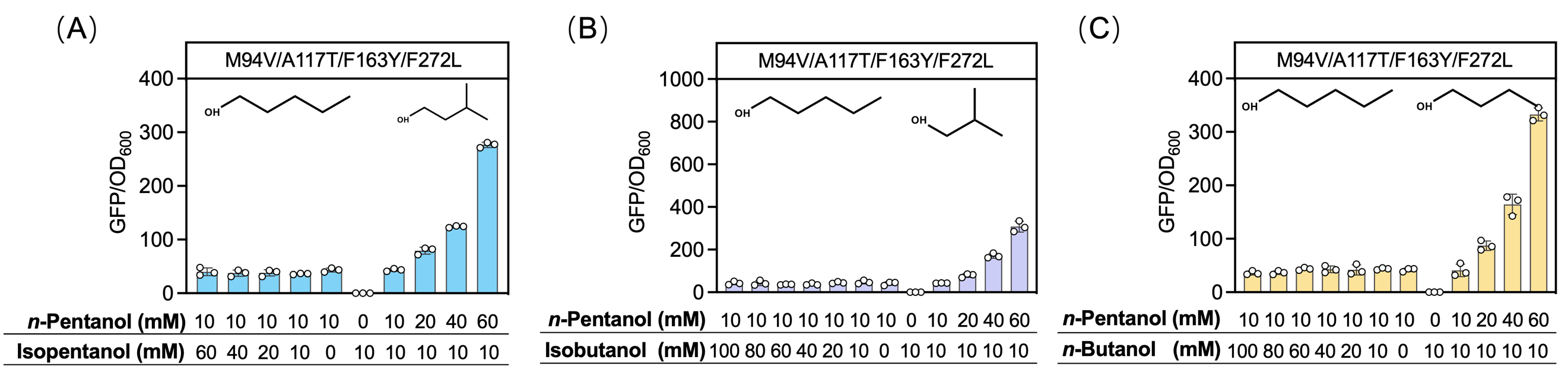
**

**Fig. S10 The response values of BmoR^M94V/A117T/F163Y/F272L^ to SM mixture.** (A) Maintaining the concentration (10 mM) of *n*-pentanol or isopentanol and increasing the concentration of *n*-pentanol or isopentanol with a gradient (0-60 mM) to confirm the orthogonality of BmoR^M94V/A117T/F163Y/F272L^ towards *n*-pentanol. (B) Maintaining the concentration (10 mM) of *n*-pentanol or isobutanol and increasing the concentration of *n*-pentanol or isobutanol with a gradient (0-60 mM or 0-100 mM) to confirm the orthogonality of BmoR^M94V/A117T/F163Y/F272L^ towards *n*-pentanol. (C) Maintaining the concentration (10 mM) of *n*-pentanol or *n*-butanol and increasing the concentration of *n*-pentanol or *n*-butanol with a gradient (0-60 mM or 0-100 mM) to confirm the orthogonality of BmoR^M94V/A117T/F163Y/F272L^ towards *n*-pentanol. Values and error bars represent mean and s.d. (n = 3), respectively.

**
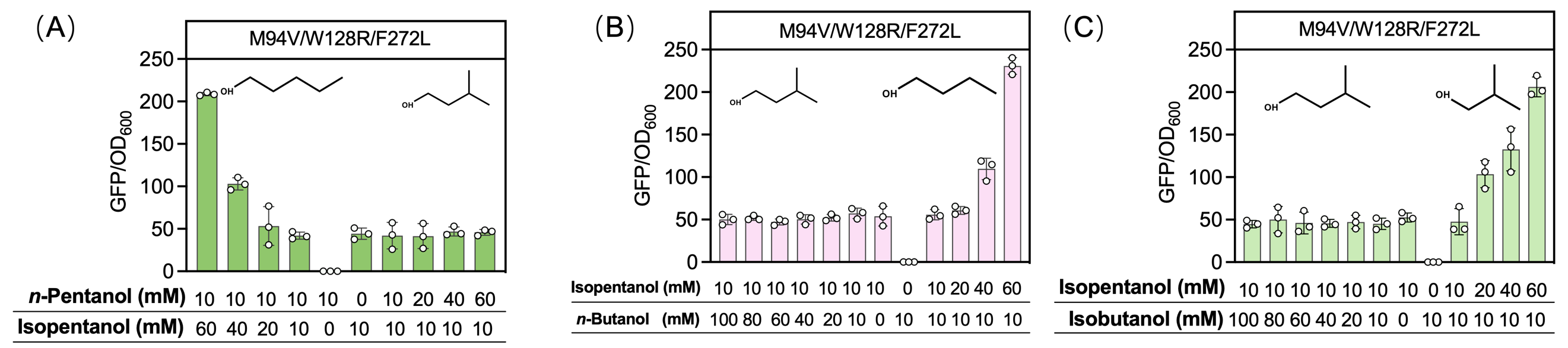
**

**Fig. S11 The response values of BmoR^M94V/W128R/F272L^ to SM mixture.** (A) Maintaining the concentration (10 mM) of *n*-pentanol or isopentanol and increasing the concentration of *n*-pentanol or isopentanol with a gradient (0-60 mM) to confirm the orthogonality of BmoR^M94V/W128R/F272L^ towards isopentanol. (B) Maintaining the concentration (10 mM) of isopentanol or *n*-butanol, and increasing the concentration of isopentanol or *n*-butanol with a gradient (0-60 mM or 0-100 mM) to confirm the orthogonality of BmoR^M94V/W128R/F272L^ towards isopentanol. (C) Maintaining the concentration (10 mM) of isopentanol or isobutanol, and increasing the concentration of isopentanol or isobutanol with a gradient (0-60 mM or 0-100 mM) to confirm the orthogonality of BmoR^M94V/W128R/F272L^ towards isopentanol. Values and error bars represent mean and s.d. (n = 3), respectively.

**
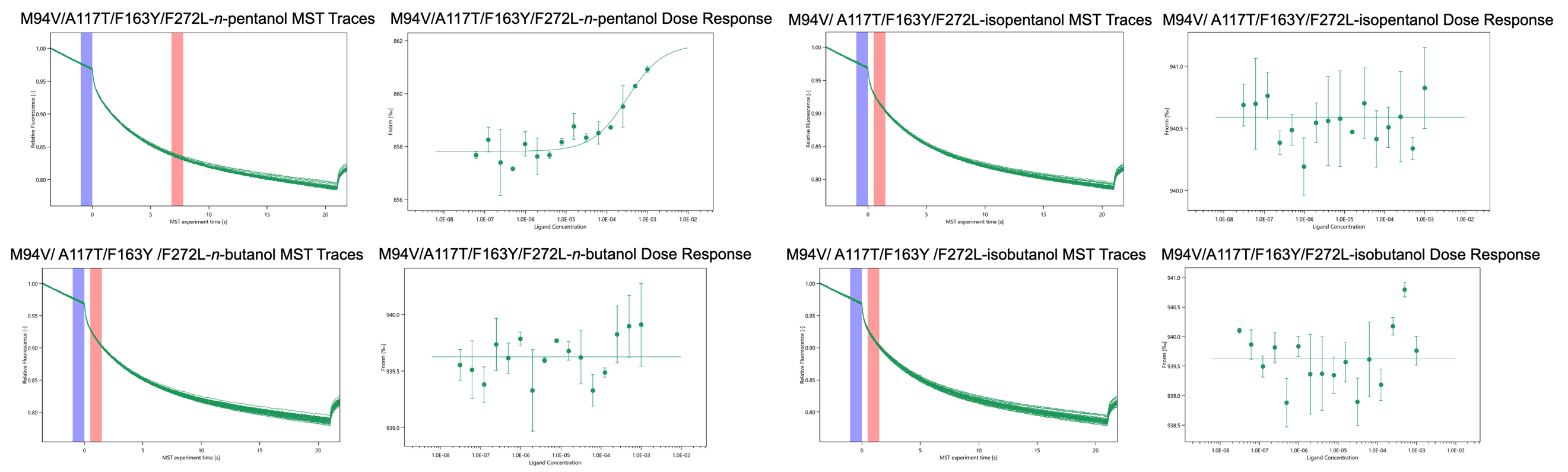
**

**Fig. S12 The fnorm fitting graphs of BmoR^M94V/A117T/F163Y/F2727L^ towards *n*-pentanol, isopentanol, *n*-butanol or isobutanol.**

**
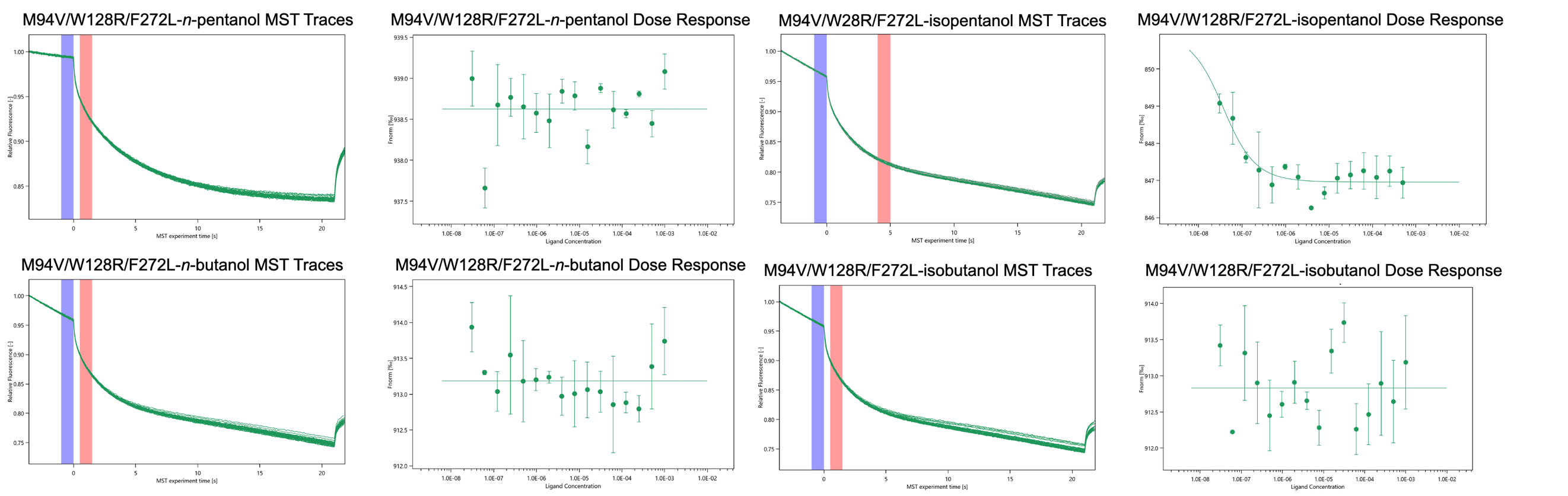
**

**Fig. S13 The fnorm fitting graphs of** **BmoR^M94V/W128R/F2727L^ towards *n*-pentanol, isopentanol, *n*-butanol or isobutanol.**

**
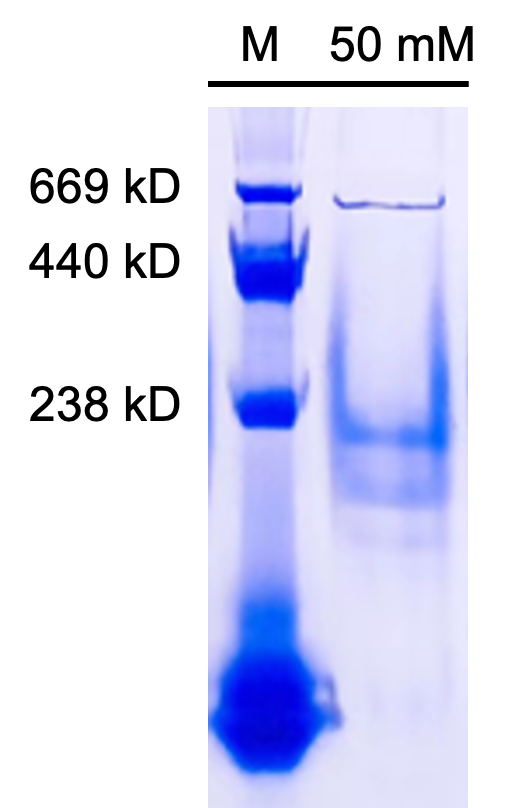
**

**Fig. S14 Non-denaturing PAGE of wild-type BmoR in presence of 50 mM *n*-butanol.** M: marker. 50 mM: wild-type BmoR in presence of 50 mM *n*-butanol.

**
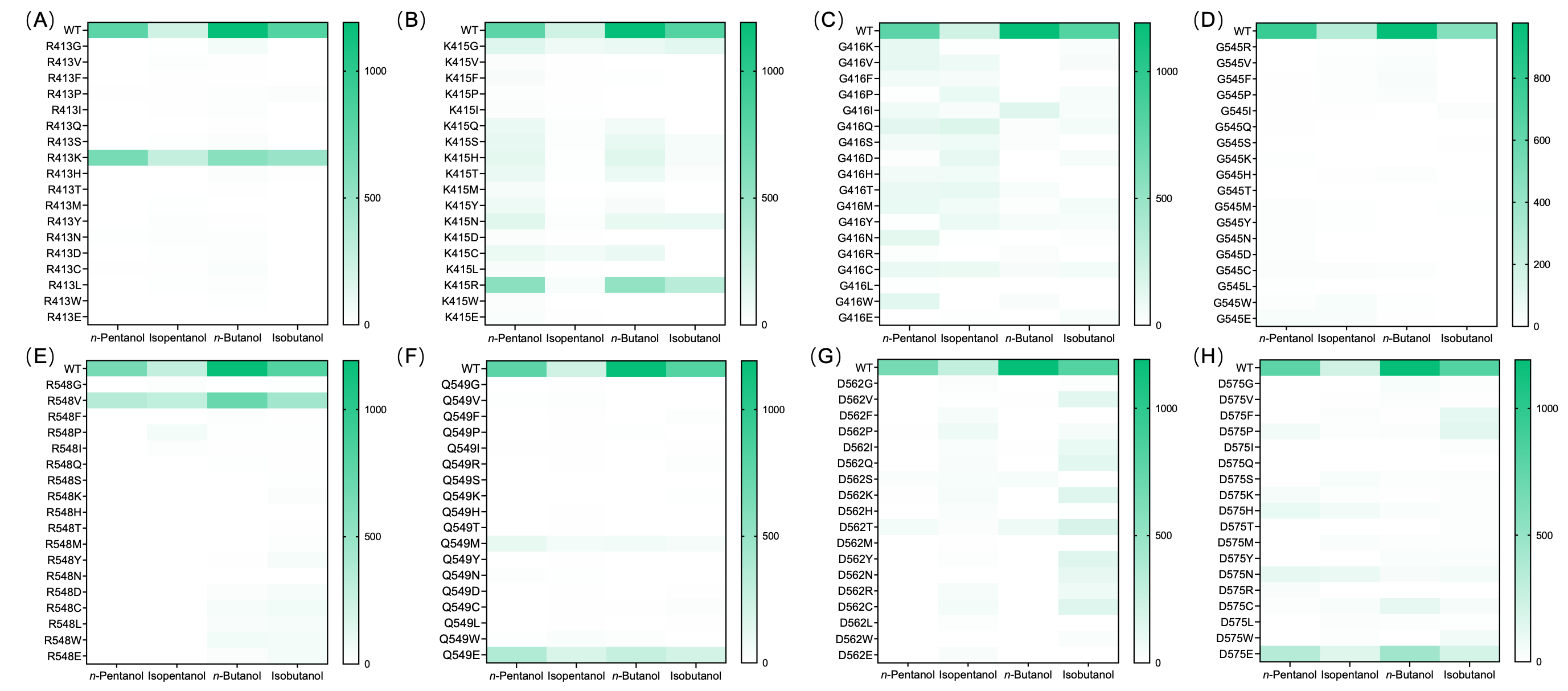
**

**Fig. S15 The response values of BmoR to four SMs after site-saturation mutagenesis.** (A) The response values of wild-type BmoR to four SMs after site-saturation mutagenesis at Arg413. (B) The response values of wild-type BmoR to four SMs after site-saturation mutagenesis at Lys415. (C) The response values of wild-type BmoR to four SMs after site-saturation mutagenesis at Gly416. (D) The response values of wild-type BmoR to four SMs after site-saturation mutagenesis at Gly545. (E) The response values of wild-type BmoR to four SMs after site-saturation mutagenesis at Arg548. (F) The response values of wild-type BmoR to four SMs after site-saturation mutagenesis at Gln549. (G) The response values of wild-type BmoR to four SMs after site-saturation mutagenesis at Asp562. (H) The response values of wild-type BmoR to four SMs after site-saturation mutagenesis at Asp575.

**Fig. S16 The response values of wild-type BmoR to four SMs after site-directed mutagenesis at Gly545 or Gly545/Glu579.**

**Fig. S17 The response values of wild-type BmoR to four SMs after site-saturation mutagenesis at Glu579.**

**Fig. S18 The OD_600_ values of the *E. coli* MG1655-derived knockout strains under the pressure of 20 mM *n*-pentanol or isopentanol.**

**Fig. S19 The OD_600_ values of MG1655 or** Δ**33 containing plasmids pWT-B^*^-LBD^*^D and pWT-SCDA^*^BCD during shake-flask fermentation.**


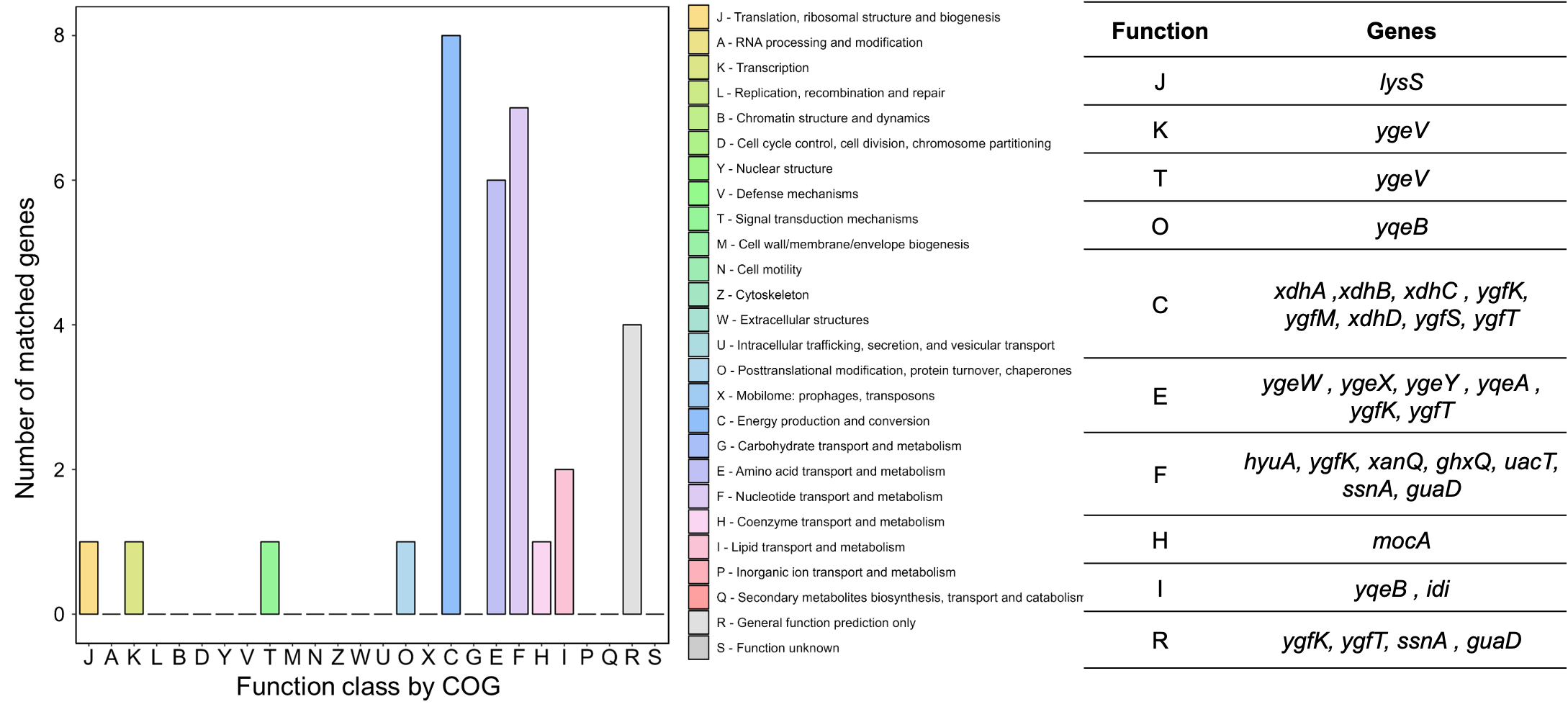


**Fig. S20 Functions of the knockout genes in strain** Δ**33.**

**
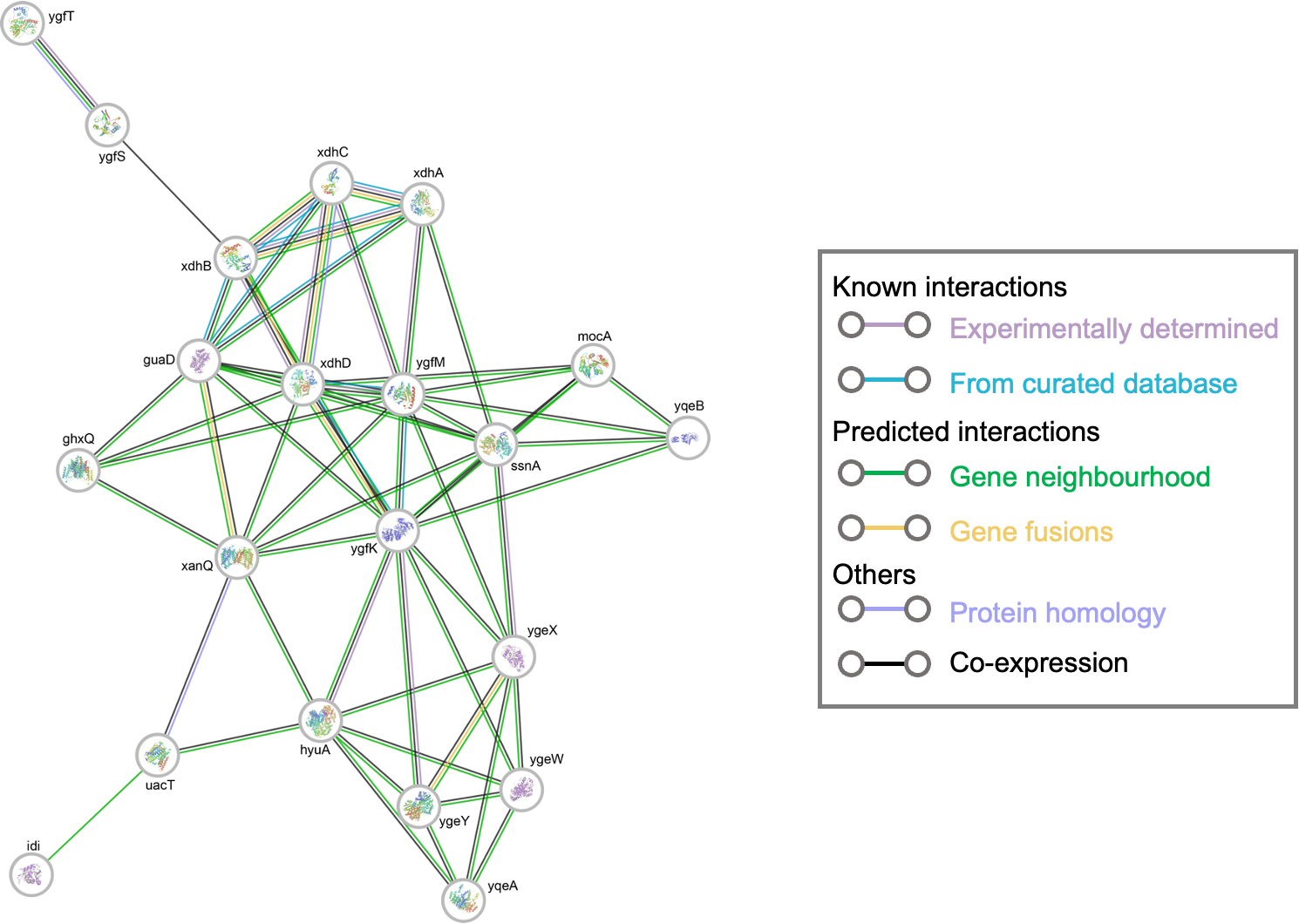
Fig. S21 Analysis of the correlation between the knockout genes in strain** Δ**33.**

**
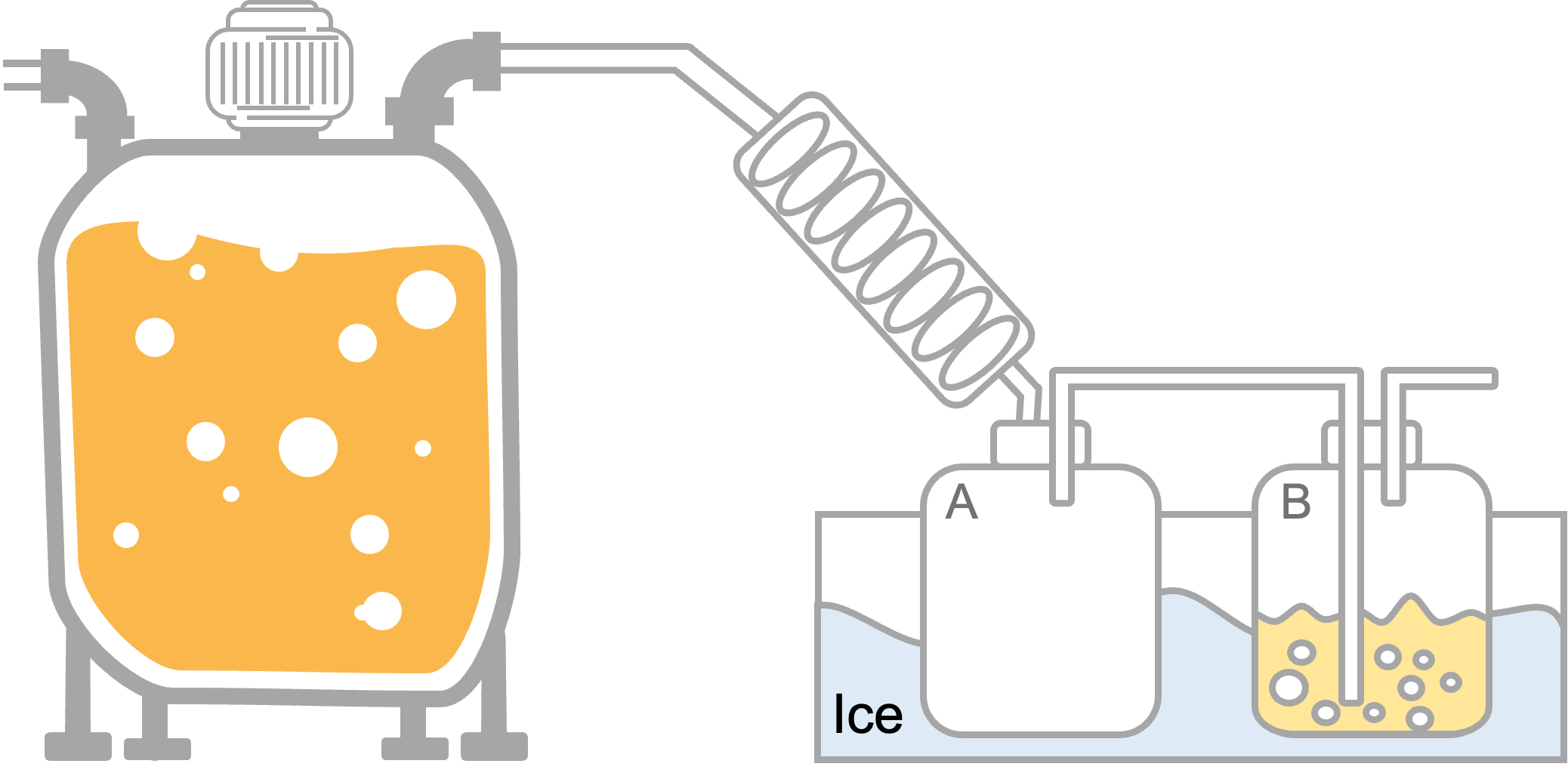
**

**Fig. S22 Bioreactor for isopentanol production.**


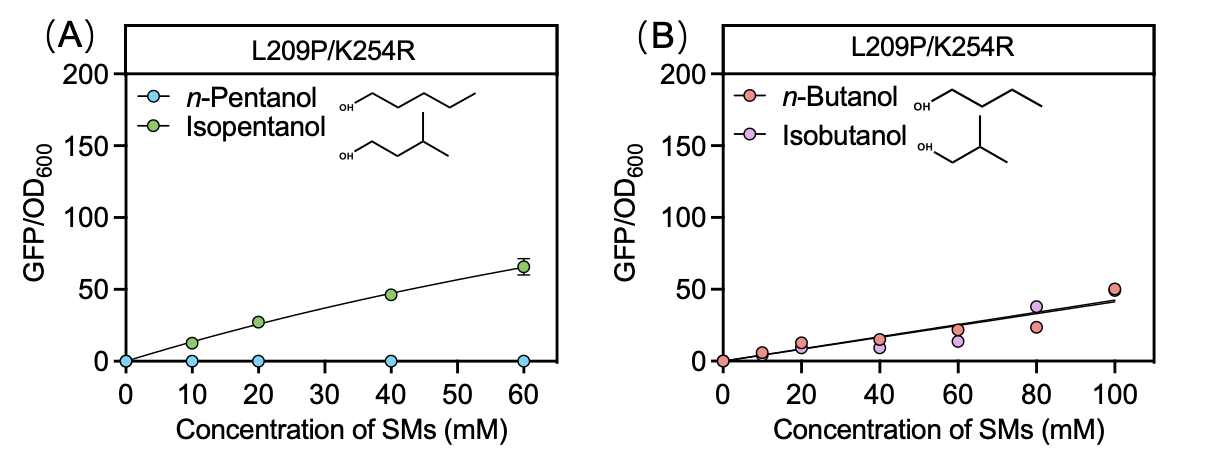


**Fig. S23 The response values of BmoR^L209P/K254R^ to *n*-pentanol, isopentanol, *n*-butanol or isobutanol.** (A) The response values of BmoR^L209P/K254R^ to *n*-pentanol or isopentanol. (B) The response values of BmoR^L209P/K254R^ to *n*-butanol or isobutanol. Values and error bars represent mean and s.d. (n = 3), respectively.

**
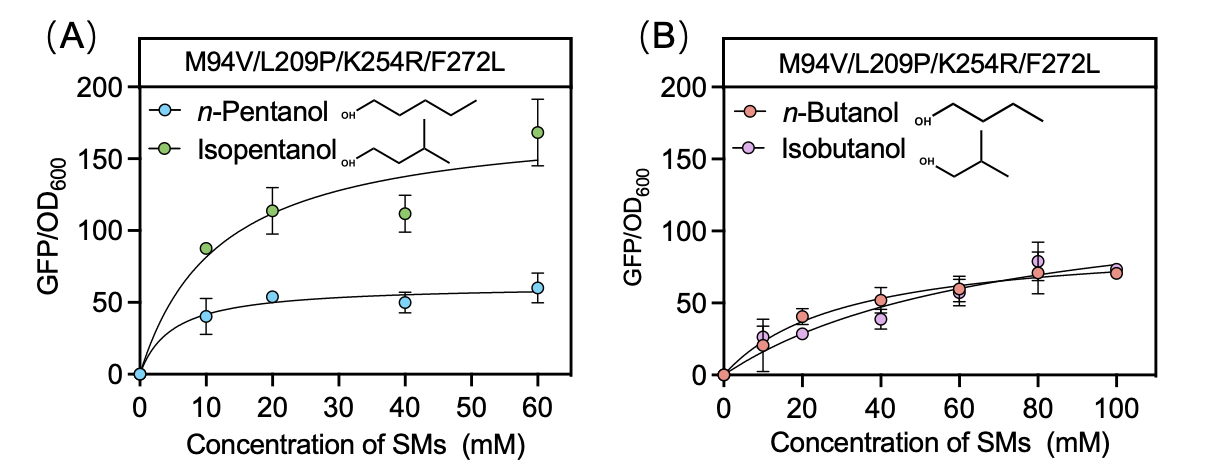
**

**Fig. S24 The response values of BmoR^M94V/L209P/K254R/F272L^ to *n*-pentanol, isopentanol, *n*-butanol or isobutanol.** (A) The response values of BmoR^M94V/L209P/K254R/F272L^ to *n*-pentanol or isopentanol. (B) The response values of BmoR^M94V/L209P/K254R/F272L^ to *n*-butanol or isobutanol. Values and error bars represent mean and s.d. (n = 3), respectively.

**
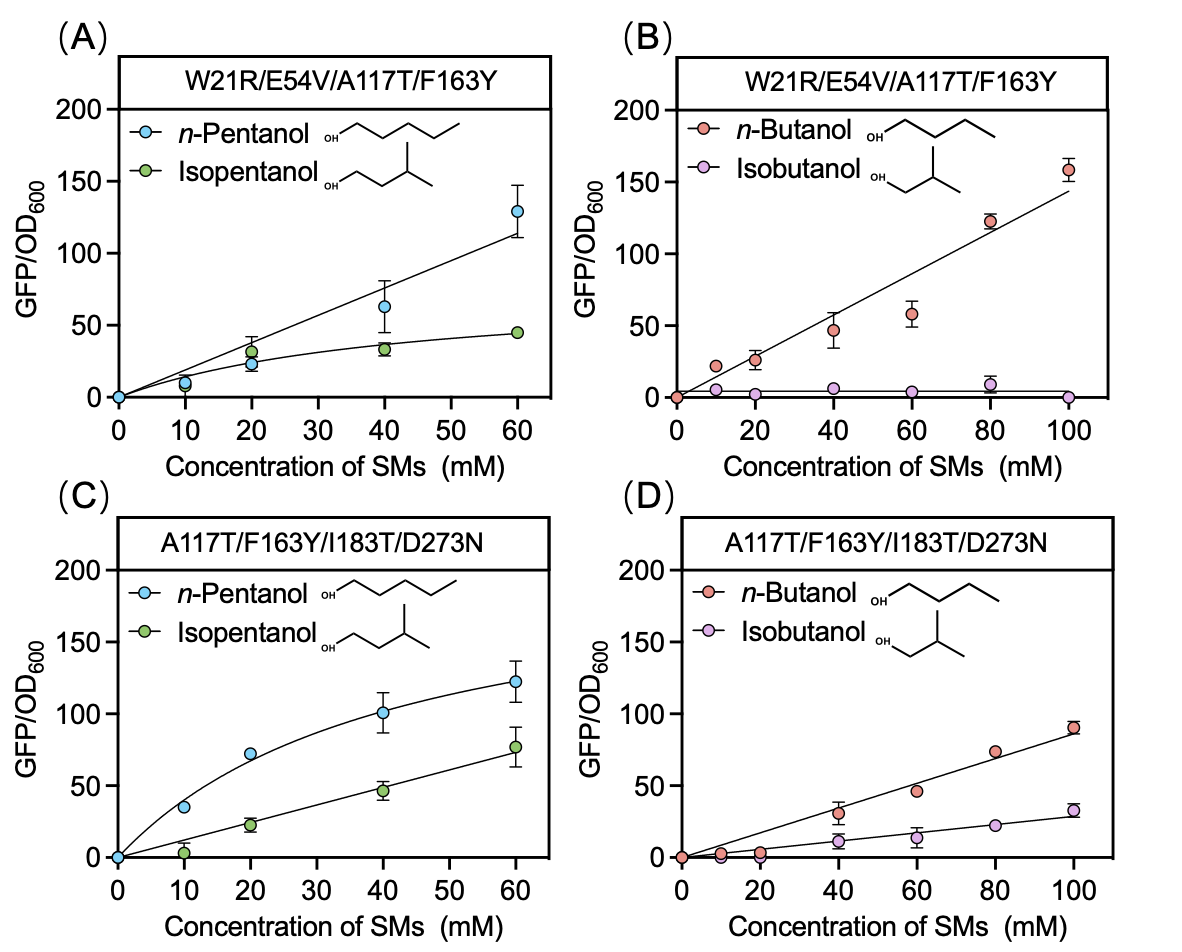
**

**Fig. S25** **The response values of BmoR^W21R/E54V/A117T/F163Y^ and BmoR^A117T/F163Y/I183T/D273N^ to *n*-pentanol, isopentanol, *n*-butanol or isobutanol.** (A) The response values of BmoR^W21R/E54V/A117T/F163Y^ towards *n*-pentanol or isopentanol. (B) The response values of BmoR^W21R/E54V/A117T/F163Y^ towards *n*-butanol or isobutanol. (C) The response values of BmoR^A117T/F163Y/I183T/D273N^ towards *n*-pentanol or isopentanol. (D) The response values of BmoR^A117T/F163Y/I183T/D273N^ towards *n*-butanol or isobutanol. Values and error bars represent mean and s.d. (n = 3), respectively.

**Fig. S26 The titers of isopentanol and isobutanol.** Strain MG1655 containing plasmids pSA69-*leuABCD* and pCS97 was used.

**Fig. S27 The titers of isopentanol and isobutanol.** Strain MG1655 containing plasmids pWT-SCDA^*^BCD and pWT-LHD^*^D was used.

**Fig. S28 The response value of BmoR^M94V/W128R/F272L^ to isopentanol and the titer of isopentanol.**
